# Multi-Detector Characterization of Grape Seed Extract to Enable *in silico* Safety Assessment

**DOI:** 10.3389/fchem.2018.00334

**Published:** 2018-08-14

**Authors:** Vincent P. Sica, Catherine Mahony, Timothy R. Baker

**Affiliations:** ^1^Corporate Functions Analytical, The Procter & Gamble Company, Mason, OH, United States; ^2^Central Product Safety, The Procter & Gamble Company Technical Centres Ltd, Egham, United Kingdom

**Keywords:** *Vitis vinifera*, grape seed, dietary supplements, oligomeric proanthocyanidins, tannins, threshold of toxicological concern, charged aerosol detector, high resolution mass spectrometry

## Abstract

Demands for increased analytical rigor have been growing within the botanical and dietary supplement industry due to concerns relative to safety, efficacy, and quality. Adulteration, ambiguous definitions, and insufficient perspective on safety are some of the major issues that arise when selecting a botanical extract. Herein, our comprehensive analytical approach is detailed for the selection of grape seed extracts. This approach provided characterization for the constituents above a threshold of toxicological concern by subjecting the extract to UHPLC-UV-CAD-HRMS and GC-FID & GC-HRMS. Thus, constituents within a wide range of volatility were evaluated. Furthermore, the extract was compared to authenticated botanical materials to confirm that no adulteration took place and was also compared to other grape seed extract sources to confirm that the material falls within the general profile. Finally, these data were cleared via an *in silico* safety assessment based on the list of constituents above the threshold of toxicological concern.

## Introduction

**Figure 1:**
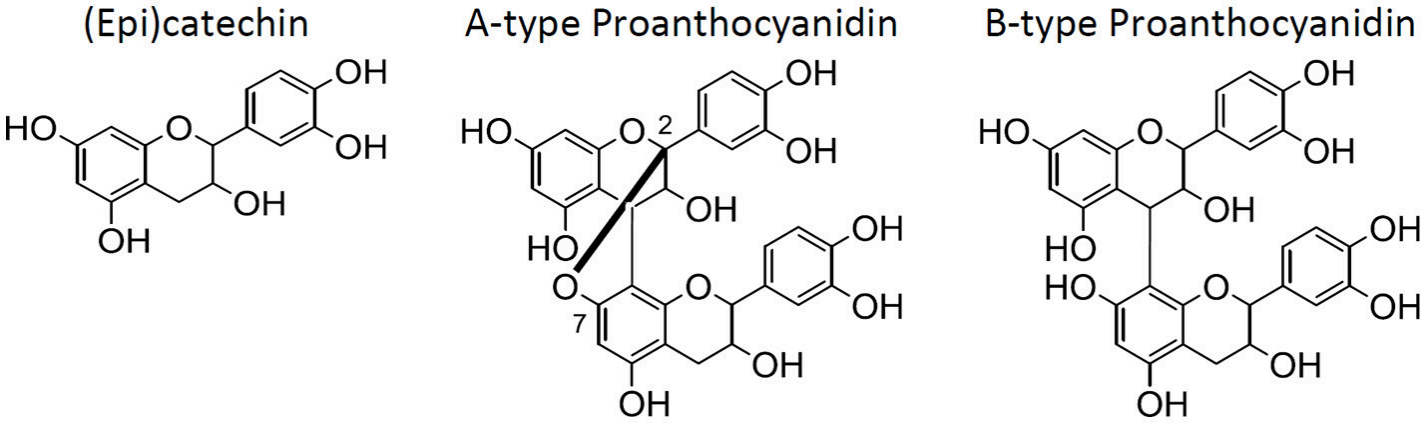
The structures for (epi)catechin, A-type proanthocyanidins, and B-type proanthocyanidins (left to right).

Grape seeds, a by-product of the juice and wine industry, are a rich source of polyphenols (Prieur et al., 1994; Labarbe et al., 1999; Peng et al., 2001; Di Lecce et al., 2014; Lin et al., 2014). Extracts of the seeds are most commonly used as an ingredient in dietary supplements due to their antioxidant potential (Aron and Kennedy, 2008; Hümmer and Schreier, 2008). Oligomeric proanthocyanidins (OPC), the class of polyphenols primarily shown to be the bioactive constituents (Aron and Kennedy, 2008; Hümmer and Schreier, 2008; Monagas et al., 2010), are polymerized (±)-catechin and (±)-epicatechin [from here onward, (epi)catechin] (Figure 1), often with galloylations. OPC are typically defined as containing 2–5 degrees of polymerization (DP), while hexamers and larger are typically categorized as tannins (≥6 DP) (Prieur et al., 1994; Labarbe et al., 1999; Peng et al., 2001; Hümmer and Schreier, 2008). This differentiation is determined by its bioavailability, since dimers to pentamers have been reported to be bioavailable (Aron and Kennedy, 2008; Hümmer and Schreier, 2008; Monagas et al., 2010). While proanthocyanidins (any degree of polymerization) are often listed as the active ingredient in grape seed extract (GSE) dietary supplements, it is ultimately the OPC content that are the most important constituents in GSE supplements. Thus, the two groups of polymerized (epi)catechins, OPC and tannins, were differentiated here onward.

The Office of Dietary Supplements (ODS—United States of America) states that safety, efficacy, and quality are the most significant concerns for botanical products (Betz, 2006). An example of these concerns is the addition of adulterants to dietary supplements. A recent survey of 21 commercial GSE products concluded that 42% of these supplements were adulterated (Villani et al., 2015). Additionally, the Botanical Adulteration Program by the American Botanical Council had issued a report detailing the issues of the addition of economic adulterants into GSE dietary supplements (Kupina and Gafner, 2016). These adulterants primarily include peanut skin and/or pine bark extracts since they also have (epi)catechin and proanthocyanidins and are less expensive than grape seeds (Kupina and Gafner, 2016). Fortunately, HPTLC, HPLC-UV, NMR, and/or mass spectrometry can readily determine whether a GSE is adulterated with peanut skin or pine bark due to the differentiation of the proanthocyanidin dimers (A-type and B-type) (Villani et al., 2015). A-type proanthocyanidins (Figure 1) are present in peanut skins and pine bark, while B-type proanthocyanidins (Figure 1) are present in grape seeds and pine bark. Since A-type proanthocyanidin dimers contain an additional C2–O–C7 bond compared to B-type proanthocyanidin dimers (Figure 1), they can be differentiated by many different analytical techniques, especially by mass spectrometry due to their difference in mass.

In addition to adulteration issues, accurate representation of a dietary supplement's contents are important quality, regulatory (label), and safety considerations. While HPLC-UV is regarded as an industrial standard for botanical analyses (i.e., quality assurance), the inherent biases of this detector can result in a misrepresentation of the compounds that lack a chromophore (e.g., sugars, fatty acids, etc.) (Bai et al., 2009; Hetrick et al., 2017). Alternatively, a universal detection system, such as a charged aerosol detector (CAD), can provide an unbiased detection system, with regard to amount of a compound, compared to UV or MS which bias toward compounds with a chromophore or ability to ionize, respectively. Instead of depending on structural properties of a constituent, the CAD uses a stream of charged gas to perform a charge transfer to the compounds and then uses an electrometer to measure the electrically charged particles (Dixon and Peterson, 2002; Eom et al., 2010). This technique allows for universal quantitative detection and the ability to calculate a response factor that is universal for all the compounds in a diverse, complex mixture. Thus, quantitation of the individual constituents in a botanical, or complex mixture, can be accurately determined without possessing a standard for each individual constituent or even class of constituents.

One shortcoming of the CAD is its inconsistency when detecting volatile and semi-volatile constituents (boiling point <400°C). Fortunately, those constituents can be analyzed using a gas chromatography-flame ionization detector (GC-FID) to provide ancillary analysis to quantitate volatile constituents, and GC-MS in both chemical ionization (CI) and electron ionization (EI) modes can be used for characterization. This ensemble of techniques can provide a comprehensive quantitative and qualitative analysis for a botanical of interest.

Herein, the individual constituents of a GSE, coded GSE-1, above a threshold of toxicological concern (TTC) were quantified and characterized using an ultrahigh performance liquid chromatography–ultraviolet–charged aerosol detector–high resolution mass spectrometry (UHPLC-UV-CAD-HRMS) system, as has been previously reported (Little et al., 2017). While there have been no indications of adverse reproductive or developmental effects in humans from dietary exposure to GSE (nor individual components that have been tested), the lack of available developmental toxicity data guided us into taking a conservative approach, using a TTC in accordance with Cramer Class III (90 μg/person/day) (European Food Safety and World Health, 2016). This allowed for a worst-case risk assessment to be performed for specific chemical entities. GSE had been tested for mutagenicity and genotoxicity both *in vitro* and *in vivo* with results pointing to no genotocicity concern (Fiume et al., 2014). Therefore, we selected a TTC fitting with the data gap that had been identified for the material, specifically developmental toxicity.

To clear a grape seed extract through the TTC approach, the CAD was used to quantify the individual constituents above the predetermined toxicological threshold. For this test case, 210 mg of GSE was the intended dose for a proposed dietary supplement formulation, which corresponded to a threshold of 400 ppm per analyte (i.e., assuming a 210 mg per day GSE exposure, 90 μg is analogous to 0.04%). Our objective was to assess and decide if the extract type, at this dose, can be supported on grounds of safety and was not to investigate the basis for efficacy. After using the CAD to quantify the constituents above the threshold, high resolution tandem mass spectrometry (MS/MS) was used to identify each of these constituents. Similarly, GC-FID and GC-HRMS were used to quantify and identify the volatile constituents, respectively. Using this multi-detector approach, the individual constituents of the GSE were characterized with the very specific goal of using the data for an *in silico* safety assessment to help guide or obviate the need for classical *in vitro* and *in vivo* safety studies (Little et al., 2017; VanderMolen et al., 2017; Baker and Regg, 2018). It should be noted that for the purposes of an *in silico* safety assessment using the TTC approach, the absolute identification of a constituent is not necessarily required. The safety approach focuses on comparing functional groups and substructures of molecules so specific connectivity is not essential. Finally, these results provided clarity of the proanthocyanidin content in GSE by comparing several sources of GSE from different suppliers, coded GSE-1 through GSE-4, and determining the extent to which the OPC and tannin contents varied between them.

## Materials and methods

### Chemical standards

The proanthocyanidin standards including the galloylated analogs were purchased from ChromaDex Inc. (Irvine, CA, USA), except for proanthocyanidin B1, which was purchased from Quality Phytochemicals LLC (East Brunswick, NJ, USA). The HPLC grade, CH_3_OH, CH_3_CN, H_2_O, and EtOH were from Honeywell (Morris Plains, NJ, USA).

### Commercial grape seed extracts

A variety of grape seed extracts were obtained from four different suppliers. GSE-1 was our chosen supplier and the focal point of this manuscript. GSE-2, GSE-3, and GSE-4 were used for comparative purposes only. Two different lots were obtained for GSE-3 and GSE-4 and both were included in the analyses.

### Authenticated reference material

The authentic grape seeds (*Vitis vinifera* L. Vitaceae) (SO SA 201601), peanut skins [*Arachis* sp.] (B), and pine bark [*Piunus pinaster* Aiton (Pinaceae)] (Nagore García Medina 201601) voucher specimens were obtained and deposited with Botanical Liaisons, LLC herbarium (Boulder, CO, USA).

### UHPLC-UV-CAD-HRMS system

The UHPLC-UV-CAD-HRMS system consisted of two Accela 1250 quaternary pumps (one for make up flow post-column), with an Accela PDA detector, split to a Corona CAD Ultra RS and Orbitrap Elite mass spectrometer (ThermoFisher, San Jose, CA, USA). The mass spectrometer was set to collect 50–2,000 *m/z* at a resolution of 60,000 at *m/z* 200. The CID fragmentation was set to 30 eV for all compounds. The voltage for positive and negative ionization modes were set to 4.0 and 3.5 kV, respectively, with a nitrogen sheath gas set to 50, an auxiliary gas at 15, and a sweep gas at 2. The S-Lens RF level was set to 62.0, and the capillary temperature was set to 360°C. The flow rate from the UHPLC was 300 μL/min using a UPLC HSS T3 (2.1 × 150 mm × 1.8 μm) column (Waters Corp., Milford, MA, USA). The mobile phase was CH_3_OH–H_2_O (acidified with 0.1% formic acid) starting at 0% CH_3_OH for 5 min, increased linearly to 50% for 50 min, then linearly to 100% for 10 min, held for 10 min, then re-equilibrated at 0% CH_3_OH. Since CAD response factors are affected by changes in the mobile phase, an inverse gradient was connected prior to splitting into the CAD and mass spectrometer to compensate for changes in the gradient. The PDA was set to acquire from 210 to 650 nm with a 9 nm resolution. The CAD was set to 100 pA and 20 pA to accurately quantitate the constituents in high and low abundances, respectively.

### GC-FID and GC-HRMS systems

The GC-FID system used was 7890A GC (Agilent, Palo Alto, CA, USA) using a HP-5MS (30 m × 0.25 mm × 0.25 μm) column. The temperature gradient was set to 40°C, held for 2 min, increased linearly at 10°C/min until 320°C, and held for 5 min. The injections were 1 μL with a 1:20 split. The GC-HRMS system used was a QExactive GC (ThermoFisher, San Jose, CA, USA) set to acquire 30–550 *m/z* using a DB-5MS (30 m × 0.25 mm × 0.25 μm) column. The temperature gradient was set to 50°C, held for 2 min, increased linearly at 10°C/min until 300°C, and held for 3 min. The injections were 1 μL with a 1:10 split. The CI gas used was methane.

### ESI-QTOF system

For the identification of the tannins, flow injection ESI-QTOF (Synapt G1, Waters, Milford, MA, USA) acquiring *m/z* 50–3,000 with collision energies of 10, 50, 70, and 110 eV was performed.

### Sample preparation for UHPLC-UV-CAD-HRMS

All of the GSEs were prepared in 70:30 CH_3_OH–H_2_O to afford a 10 mg/mL solution. The samples were vortex mixed (60 s), sonicated for 5 min, and vortex mixed (60 s) again. The samples dissolved fully and were not processed further.

### Sample preparation for GC/FID and GC/HRMS

GSE-1 was prepared in methanol to afford a 3 mg/mL solution. The sample was vortex mixed (60 s), sonicated for 5 min, vortex mixed (60 s) again. The sample dissolved fully and was not processed further. A 1.0 mg/mL hydrocarbon standard mix (Resktek, DRO mix, Lot#A0108725), comprised of hydrocarbon chains from 10 to 25 carbons in length, were diluted by a factor of 10 to a concentration of 0.1 mg/mL in dichloromethane. This standard mix was used to determine the Kovats retention indexes and the response factors were calculated based on the integration of the area under the curve of each standard in the mix. An additional standard mix was prepared using hexane (C6), heptane (C7), and nonane (C9) at a concentration of 0.1 mg/mL each and was used to calculate the Kovats retention indexes for the early eluting compounds.

### Sample preparation for authenticated materials

Authenticated grape seeds (*V. vinifera*), peanut skins (*Arachis* sp.), and pine bark (*P. pinaster*) were separately ground using a mortar and pestle until the samples were a powder and the larger particulates were removed. Then, aliquots (1 g) of each powder were weighed into separate 20-mL glass scintillation vials followed by addition of 10.0 mL of 70:30 EtOH–H_2_O to each vial. The samples were vortex mixed (60 s), sonicated for 30 min, vortex mixed (60 s) again and then filtered through 0.45 μm filters to remove the solid particulates. They were then evaporated to dryness using streams of nitrogen air. The dried products were reconstituted in 70:30 CH_3_OH–H_2_O at 10 mg/mL for the comparative analyses.

### Treatment of sample with poly(vinylpolypyrrolidone)

The GSE-1 (5 mg) was weighed into a 2 mL autosampler vial and 1.00 mL of 70:30 CH_3_OH–H_2_O was added. Poly(vinylpolypyrrolidone) (PVPP) (10 mg) was weighed into another 2 mL autosampler vial followed by the addition of 0.800 mL of 70:30 CH_3_OH–H_2_O. Subsequently, 0.200 mL of this grape seed solution was added to the original vial containing PVPP. The sample was vortex mixed (5 min) then placed in the centrifuge at 3000 rcf for 5 min. The supernatant was transferred to a new autosampler vial and ready for analysis.

### Treatment of sample with molecular weight cut-off (MWCO) filters

The GSE-1 was prepared in 70:30 CH_3_OH–H_2_O to afford a 10 mg/mL solution. The solution was transferred into the MWCO filter tube of choice (3, 10, 30, 50, or 100 K). The tubes were placed into the centrifuge at 14,000 rcf for 30 min. The retentates were reconstituted in 70:30 CH_3_OH–H_2_O and re-centrifuged in triplicate.

### Sample preparation for ESI-QTOF

The GSE-1 was prepared in 70:30 CH_3_OH–H_2_O to afford a 10 mg/mL solution. The solution was transferred into the 30,000 MWCO filter tube. The tube was placed into the centrifuge at 14,000 rcf for 30 min. The retentate was reconstituted in 70:30 CH_3_OH–H_2_O and centrifuged again. This process was performed a total of six times since it was observed that some OPC remained when only performed in triplicate. The final retentate was transferred to a new 4 mL vial and evaporated to dryness. The dried retentate was reconstituted in methanol to 1 mg/mL concentration and subjected to flow injection ESI-QTOF.

### Standard preparation

Individual stock solutions were prepared at 1.0 mg/mL in 70:30 CH_3_OH–H_2_O for catechin (monomer), epicatechin (monomer), proanthocyanidin B2 (dimer), and proanthocyanidin C1 (trimer). A combined high standard (100 μg/mL each component) was prepared by adding 0.100 mL of each polyphenol to 0.600 mL of 70:30 CH_3_OH–H_2_O. Standards at 20, 4.0, and 0.8 μg/mL were prepared in 70:30 CH_3_OH–H_2_O by serial dilution. The tannins standard at 10,000, 1,000, and 100 μg/mL were prepared in 70:30 CH_3_OH–H_2_O by serial dilution.

## Results and discussion

### Adulteration

**Figure 2:**
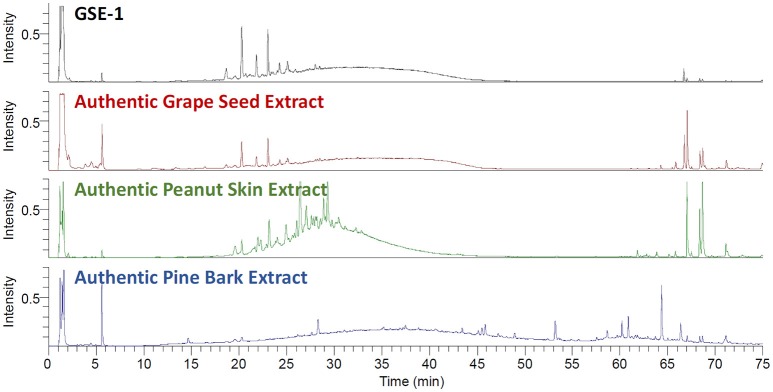
The stacked UHPLC-CAD chromatograms of the GSE-1 extract, the extracted authentic grape seed (*Vitis vinifera*) material, the extracted authentic peanut skin (*Arachis sp*.) material (green trace), and the extracted authentic pine skin (*Piunus pinaster*) material (blue trace). The GSE-1 and authentic grape seed extracts were in agreement. There were no indications of adulteration in the GSE-1 extract when compared to the peanut skin and pine bark traces.

The first step in evaluating a GSE supplier for use in a dietary supplement was to determine whether the botanical extract was adulterated. For GSE dietary supplements, the two most prominent economic adulterants of concern are peanut skins and pine bark extracts (Villani et al., 2015). Authentic voucher specimen of grape seeds (*V. vinifera*), peanut skins (*Arachis* sp.), and pine bark (*P. pinaster*) were obtained, extracted, and analyzed using the UHPLC-UV-CAD-HRMS system. The CAD chromatograms were compared to determine if there were any signs of adulteration in the GSE-1 (Figure 2). The GSE-1 was essentially equivalent to the authentic GSE voucher, while being noticeably different from the peanut skins and pine bark extracts. The CAD results were further supported by negative ionization mode MS and UV chromatographic traces (Figures S1, S2).

Previously, TLC and/or NMR have been used to determine the presence of adulterants in GSE (Villani et al., 2015). However, the UHPLC-UV-CAD-HRMS system could confidently confirm that there was no adulteration to the GSE-1 with an even greater level of sensitivity. The HRMS data was filtered for key adulterant constituents (e.g., A-type proanthocyanidins) using narrow mass chromatograms since A-type proanthocyanidins (*m/z* 575.1189) in negative ionization mode can be easily differentiated from B-type proanthocyanidins (*m/z* 577.1345). The HRMS data confirmed that GSE-1 was not adulterated with the peanut skins nor pine bark by a comparison of proanthocyanidin A1 and A2 standards with GSE-1 (Figure S3).

Additionally, based on the CAD chromatograms, there was only one obvious CAD peak at 23.36 min in the GSE-1 that was not in the authentic GSE (Figure S4). The HRMS signals corresponding to the CAD peak displayed *m/z* 291.0873 in negative ionization mode was assigned the formula of C_15_H_15_O_6_ (calculated. for C_15_H_15_O_6_, 291.0874, −0.4 ppm). This constituent was identified as 1-(3′,4′-dihydroxyphenyl)-3-(2″,4″,6″-trihydroxyophenyl)propan-2-ol by matching the MS/MS spectrum to literature (Sánchez-Patán et al., 2012). This compound (Figure S4) is a known catabolized product of (epi)catechin (Sánchez-Patán et al., 2012) and metabolized product of proanthocyanidin dimers (Appeldoorn et al., 2009). With (epi)catechin and proanthocyanidin dimers being prevalent in the sample, it may be a potential low-level degradation product during the processing of the GSE-1. Additionally, another potential degradant, a proanthocyanidin dimer-like compound, was detected at trace levels (below the TTC) at 27.37 min in the sample. This compound appeared to have undergone a similar reductive cleavage of the heterocyclic C-ring of one of the flavan-3-ols from a proanthocyanidin dimer (Figure S5). While the structure of this compound has not been reported, interpretation of the MS/MS spectra supported the proposed structure (Figure S5) (Köhler et al., 2008).

### Quantitation and characterization by UHPLC-CAD-UV-MS/MS

The CAD was also used to quantify the amount of the constituents in the GSE-1. Catechin, epicatechin, proanthocyanidin B2 (dimer), and proanthocyanidin C1 (trimer) were used as standards to calculate a response factor from the CAD, to quantitate all the peaks in the sample chromatogram. In total, a mass balance of 91% was determined. Thus, 91% of GSE-1 bulk material was accounted for by the CAD detector. Furthermore, the response factor was used to determine which CAD peaks were above the threshold of toxicological concern (Little et al., 2017; i.e., at 90 μg per 210 mg dose) for the GSE dietary supplement. Ultimately, there were a total of 39 CAD peaks detected, which were comprised of at least 83 individual components, as determined by HRMS (Figure S6). The 39th “peak” was attributed to the broad peak in the chromatogram and was identified as tannins (polymerized catechin with ≥6 DP). It is interesting to note that the tannin hump observed for the three extracts (Figure 2), GSE, pine bark and peanut skin, are markedly distinct.

**Figure 3:**
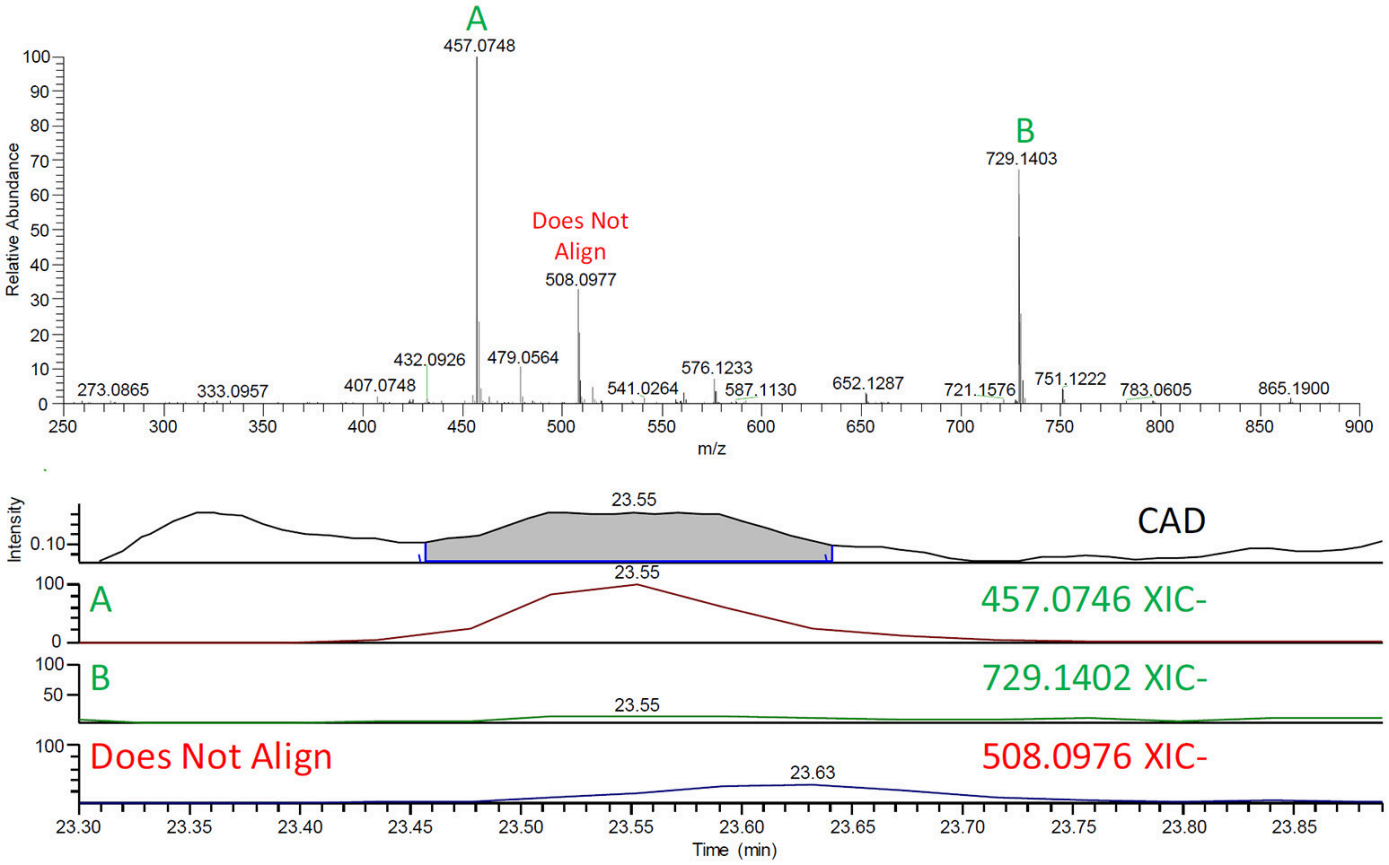
**(Top)** The negative mode mass spectrum at 23.55 min (CAD Peak# 24). **(Bottom)** The CAD chromatogram and subsequent exact mass chromatograms (XIC) of the masses >20% from the spectrum. Note that *m*/*z* (A) 457 and (B) 729 align under the CAD signal while *m*/*z* 508 has a slightly delayed retention time and does not align with the CAD. Since corresponding MS and CAD signals have the same retention time, *m*/*z* 508 was not identified as a contributor to the CAD signal.

In several cases, multiple components, as indicated by HRMS, co-eluted under one CAD peak. To determine the contributing analytes, exact mass chromatograms were generated for all the *m/z* signals >20% intensity in the summed mass spectra, in both positive and negative ionization modes, aligned under the CAD peak (Figure 3). The *m/z* signals that did not align by retention time were not considered to be contributors to the CAD signal, while the masses that did align were then subsequently identified using MS/MS (Table 1, Table S1).

**Table 1:** Proposed identifications of components producing CAD peaks in the UHPLC-UV-CAD-HRMS analysis of GSE-1.

**CAD Peak**	**RT (min)**	**Proposed Identification**	**Molecular Formula**	**Amt. μg per 210 mg dose**	**Confidence (A-D)**	**Accurate mass matches formula**	**MS/MS matches structure**	**Literature/database match**	**Reference match**
1a	1.04	Magnesium salts	Mg_x_Fm_y_	700	Partial	✓ (E)			
1b		Calcium salts	Ca_x_Fm_y_		Partial	✓ (E)			
2a	1.18	Sodium salts	Na_x_Fm_y_	4,600	Partial	✓ (E)			
2b		Potassium salts	K_x_Fm_y_		Partial	✓ (E)			
3a	1.37	Monosaccharide	C_6_H_12_O_6_	16,000	Matched	✓	✓ (F)	✓ Eyduran et al., 2015; Musingarabwi et al., 2016	
3b		Gluconic Acid	C_6_H_12_O_7_		Matched	✓	✓	✓ Larcher et al., 2009; 2017c	
3c		Glutamic Acid	C_5_H_9_NO_4_		Reference	✓	✓	✓ Bouloumpasi et al., 2002	✓
3d		Choline hexoside	C_11_H_23_NO_6_		Tentative	✓	✓(G)	✓ Eyduran et al., 2015	
3e		Arginyl fructose	C_12_H_24_N_4_O_7_		Tentative	✓	✓	✓ Ryu et al., 2001; Joo et al., 2008	
3f		Cellobiosan	C_12_H_20_O_10_		Partial	✓	✓(G)	Hurt et al., 2013	
3g		Isovaline	C_5_H_11_NO_2_		Matched	✓	✓	✓^2016^	
4a	1.53	Tartaric Acid	C_4_H_6_O_6_	5,700	Matched	✓	✓	✓ Eyduran et al., 2015; Musingarabwi et al., 2016; 2017c	
4b		Disaccharide	C_12_H_22_O_11_		Matched	✓	✓	✓ Eyduran et al., 2015; Musingarabwi et al., 2016; 2017c	
4c		Proline	C_5_H_9_NO_2_		Reference	✓	✓	✓ Bouloumpasi et al., 2002	
4d		N-methylnicotinate	C_7_H_7_NO_2_		Matched	✓	✓	✓ Eyduran et al., 2015; Musingarabwi et al., 2016	
5a	2.17	Malic Acid	C_4_H_6_O_5_	1,700	Reference	✓	✓	✓ Musingarabwi et al., 2016	✓
5b		*Unidentified*	–		Unknown		(H)		
6a	4.46	Citric Acid	C_6_H_8_O_7_	51	Reference	✓	✓	✓ Eyduran et al., 2015	✓
6b		Pyroglutamic Acid	C_6_H_8_O_7_		Matched	✓	✓	✓ 2017c	
7a	5.56	Succinic Acid	C_4_H_6_O_4_	540	Reference	✓	✓	✓	
7b		Tyrosine	C_9_H_11_NO_3_		Reference			Eyduran et al., 2015	
7c		Uridine	C_9_H_12_N_2_O_6_		Matched	✓	✓	✓2017c	
7d		5′-O-(β-D-Glucopyranosyl) pyridoxine	C_14_H_21_NO_8_		Tentative	✓	✓ (G)	Nikolić et al., 2012	
7e		Adenosine	C_10_H_14_N_5_O_4_		Matched	✓	✓	✓ 2017c	
7f		Leucine-Fructose	C_12_H_23_O_7_		Tentative	✓	✓		
7g		*Unidentified*	C_9_H_16_O_8_		Partial	✓			
7h		*Unidentified*	C_9_H_18_O_8_		Partial	✓			
7i		*Unidentified*	C_10_H_14_O_8_N_2_		Partial	✓			
7j		*Unidentified*	C_11_H_21_N_2_O_3_		Partial	✓			
7k		*Unidentified*	C_12_H_21_N_2_O_3_		Partial	✓			
7l		*Unidentified*	C_19_H_12_N_3_O_2_		Partial	✓			
8	9.46	Gallic acid	C_7_H_6_O_5_	210	Reference	✓	✓	✓ Lin et al., 2014	✓
9a	13.39	Glucogallin	C_13_H_16_O_10_	180	Matched	✓	✓	✓ Li et al., 2016	
9b		*Unidentified*			Unknown	(I)			
10	16.39	Tryptophan	C_11_H_12_N_2_O_2_	92	Reference	✓	✓	✓ Bouloumpasi et al., 2002	✓
11	18.62	Proanthocyanidin B1	C_30_H_26_O_12_	1,400	Reference	✓	✓	✓ Lin et al., 2014	✓
12	19.02	Proanthocyanidin B	C_30_H_26_O_12_	110	Matched	✓	✓	✓ Lin et al., 2014	
13	19.57	Proanthocyanidin B	C_30_H_26_O_12_	510	Matched	✓	✓	✓ Lin et al., 2014	
14	20.25	Catechin	C_15_H_14_O_6_	4,100	Reference	✓	✓	✓ Lin et al., 2014	✓
15a	20.57	Proanthocyanidin B	C_30_H_26_O_12_	83	Matched	✓	✓	✓ Lin et al., 2014	
15b		Gallocatechin Gallate isomer	C_22_H_18_O_11_		Tentative	✓	✓(J)	✓ Lin et al., 2014	
15c		*Unidentified*			Unknown	(K)			
16	20.69	Proanthocyanidin C	C_45_H_38_O_18_	180	Matched	✓	✓	✓ Lin et al., 2014	
17	21.01	Benzyl alcohol	C_18_H_26_O_10_	97	Tentative	✓	✓(G)	Amessis-Ouchemoukh et al., 2014	
18	21.14	Galloylated Proanthocyanidin (tetramer)	C_67_H_54_O_28_	120	Matched	✓	✓	✓ Lin et al., 2014	
19	21.82	Proanthocyanidin B2	C_30_H_26_O_12_	1,700	Reference	✓	✓	✓ Lin et al., 2014	✓
20a	22.39	Proanthocyanidin B	C_30_H_26_O_12_	180	Matched	✓	✓	✓ Lin et al., 2014	
20b		Lariciresinol Glucoside analog	C_26_H_34_O_11_		Tentative	✓	✓(L)	Baderschneider and Winterhalter, 2001	
20c		*Unidentified*	C_13_H_14_O_3_		Partial	✓	(M)		
21	22.73	Proanthocyanidin B	C_30_H_26_O_12_	87	Matched	✓	✓	✓ Lin et al., 2014	
22	23.02	Epicatechin	C_15_H_14_O_6_	3,500	Reference	✓	✓	✓ Lin et al., 2014	✓
23a	23.36	1-(3′,4′-dihydroxy-phenyl)-3-(2″,4″,6″-trihydroxyophenyl) propan-2-ol	C_15_H_16_O_6_	180	Matched	✓	✓	✓ Appeldoorn et al., 2009; Sánchez-Patán et al., 2012	
23b		Dihydrokaempferol 3-O-ß-D-glucoside	C_21_H_22_O_11_		Matched	✓	✓	✓ Pati et al., 2014	
24a	23.54	Epigallocatechin Gallate	C_22_H_18_O_11_	270	Reference	✓	✓	✓ Lin et al., 2014	✓
24b		Galloylated Proanthocyanidin (dimer)	C_37_H_30_O_16_		Matched	✓	✓	✓ Lin et al., 2014	
24c		Proanthocyanidin (tetramer)	C_60_H_50_O_24_		Matched	✓	✓	✓ Lin et al., 2014	
25	23.99	Proanthocyanidin C	C_45_H_38_O_18_	240	Matched	✓	✓	✓ Lin et al., 2014	
26a	24.24	Proanthocyanidin C1	C_45_H_38_O_18_	890	Reference	✓	✓	✓ Lin et al., 2014	✓
26b		Leptolepisol D	C_27_H_32_O_10_		Tentative	✓	✓	Liu et al., 2016
27a	24.94	Proanthocyanidin B	C_30_H_26_O_12_	230	Matched	✓	✓	✓ Lin et al., 2014	✓
27b		Galloylated Proanthocyanidin (trimer)	C_52_H_42_O_22_		Matched	✓	✓	✓ Lin et al., 2014	
28a	25.07	Galloylated Proanthocyanidin (dimer)	C_37_H_30_O_16_	950	Matched	✓	✓	✓ Lin et al., 2014	
28b		Proanthocyanidin (tetramer)	C_60_H_50_O_24_		Matched	✓	✓	✓ Lin et al., 2014	
29a	25.30	Galloylated Proanthocyanidin (trimer)	C_52_H_42_O_22_	85	Matched	✓	✓	✓ Lin et al., 2014	
29b		Lariciresinol Glucosides analog	C_26_H_34_O_11_		Tentative	✓	✓(L)	Baderschneider and Winterhalter, 2001	
30a	25.91	Galloylated Proanthocyanidin (trimer)	C_52_H_42_O_22_	240	Matched	✓	✓	✓ Lin et al., 2014	
30b		Proanthocyanidin (pentamer)	C_75_H_62_O_30_		Matched	✓	✓	✓ Lin et al., 2014	
31	26.32	Proanthocyanidin B	C_30_H_26_O_12_	93	Matched	✓	✓	✓ Lin et al., 2014	
32a	27.37	Galloylated Proanthocyanidin (trimer)	C_52_H_42_O_22_	110	Matched	✓	✓	✓ Lin et al., 2014	
32b		Rutin	C_27_H_30_O_16_		Reference	✓	✓	✓ Iacopini et al., 2008	
32c		Galloylated Proanthocyanidin (dimer)	C_44_H_34_O_20_		Matched	✓	✓	✓ Lin et al., 2014	
32d		2-(3,4-dihydroxyphenyl)-4-(3-(3-(3,4-dihydroxyphenyl)-2-hydroxypropyl)-2,4,6-trihydroxylphenyl) chromane-3,5,7-triol	C_30_H_28_O_12_		Tentative	✓	✓(N)	Köhler et al., 2008	
32e		Methylated Proanthocyanidin B-type analog	C_31_H_28_O_12_		Tentative	✓	✓	Lin et al., 2014	
33a	28.00	Epicatechin gallate	C_22_H_18_O_10_	320	Reference	✓	✓	✓ Lin et al., 2014	✓
33b		Proanthocyanidin B	C_30_H_26_O_12_		Matched	✓	✓	✓ Lin et al., 2014	
34a	28.41	Catechin gallate	C_22_H_18_O_10_	100	Reference	✓	✓	✓ Lin et al., 2014	✓
34b		Galloylated Proanthocyanidin (trimer)	C_52_H_42_O_22_		Matched	✓	✓	✓ Lin et al., 2014	
34c		Embigenin	C_23_H_24_O_10_		Tentative	✓	✓ (G)	Bakhtiar et al., 1994	
35	66.70	Ursolic Acid	C_30_H_48_O_3_	690	Tentative	✓	✓ (O)	✓ 2017c	
36	67.04	Linoleic Acid	C_18_H_32_O_2_	200	Matched	✓	✓	✓ 2017c	
37	68.37	Palmitic Acid	C_16_H_32_O_2_	170	Matched	✓	✓	✓ 2016	
38	68.66	Oleic Acid	C_18_H_34_O_2_	110	Matched	✓	✓	✓2016; 2017c	
39	15-45	Tannins		~160,000	Matched	✓	✓	✓ Peng et al., 2001	

*^A^**Partial:** The proposed identification has a molecular formula that matches in terms of accurate mass*.

*^B^**Tentative:** The proposed identification matches in terms of accurate mass and interpretation of MS/MS*.

*^C^**Matched:** The proposed identification matches a literature reference and/or online database [ (2017c) or (2016)] in terms of accurate mass and MS/MS*.

*^D^**Reference:** The proposed identification matches a reference standard in terms of retention time, accurate mass, and tandem mass spectrometry (MS/MS)*.

*^E^Observed peaks for metal (M) clusters with formate (Fm) and acetonitrile (ACN) from the mobile phase. For example, [MFm + ACN]+ in positive-mode and [MFm3]– in negative-mode. It is believed that the anions are exchanging in solution (e.g., Cl- Fm-). The original anion is unknown. Common observance in other botanicals*.

*^F^Identified as a monosaccharide but could not be further narrowed down by UHPLC-MS/MS*.

*^G^The exact position and/or identity of the aglycone cannot be determined by UHPLC-MS/MS*.

*^H^HRMS data was unable to assign formula, but positive-ion MS/MS data displays a loss of malic acid and the chromatographic trace follows exactly with malic acid standard*.

*^I^HRMS data unable to assign formula, but chromatographic data follows exactly with glucogallin, even under different chromatographic conditions*.

*^J^HRMS data consistent with formula. MS/MS data does not match with the reference standard with the same formula, but does contain fragments indicating it is related to the class of compounds. The gallate is believed to be on one of the remaining hydroxy groups of the A-ring based on interpretation of MS/MS*.

*^K^HRMS data unable to assign formula*.

*^L^Literature reports another compound with same formula and matching MS/MS spectra. Could not differentiate between the two without a standard but the alternative structure was assigned to peak 29b/20b*.

*^M^There are several peaks (>10) matching this accurate mass throughout the run with identical MS/MS. Peaks are sharp, indicating that they aren't just background noise, but this is the only retention time where it is under a CAD peak of significance. Not believed to be anything of significant contribution to the CAD signal*.

*^N^HRMS data consistent with formula. Negative-ion MS/MS similar to known compound but does not match [34]. Proposed structure is based on MS/MS interpretation*.

*^O^The exact position of some functional groups cannot be determined by UHPLC-MS/MS*.

To utilize this data in an *in silico* toxicological safety assessment, the analyte identifications were categorized with levels of confidence: **reference**—if a reference standard matched in retention time, accurate mass, and MS/MS fragmentation pattern; **matched**—if the accurate mass and MS/MS match literature or online databases [ (2017c) or (2016)]; **tentative**—if the accurate mass and MS/MS match supported structure but data from literature or online databases are limited; **partial**—if accurate mass provided a molecular formula but no complete structure can be assigned; **unknown**—if a reasonable molecular formula cannot be derived from the accurate mass. Of the 83 constituents, 17 were confirmed by a **reference** standard, 39 **matched** literature, 12 were **tentatively** identified, 12 were assigned a molecular formula, and 3 remained unidentified (Table 1, Table S1). The three unknown constituents were all under CAD peaks along with multiple other mass spectrometry signals and were estimated to be individually well below the threshold of toxicological interest. Once again, definitive identifications were not necessarily required to pass the *in silico* safety assessment. Even the partial identifications add value to this assessment and the ability to conclude a botanical is of low toxicity concern. These data were tabulated and organized in order of retention time, then assigned a peak number based on the CAD chromatogram. The constituents were identified to a level of confidence and quantified to the amount (μg) per supplement dose (210 mg) using the response factor from the CAD (Table 1, Table S1). Finally, these data were sent for an internal toxicological review, which has been summarized below.

The constituents were categorized into three types of compounds: Polar, nonpolar, and polyphenols. Polar compounds [salts, amino acids, organic acids (e.g., malic acid), and sugars] made up 16% of the total CAD signal. Nonpolar compounds [fatty acids and sterols] were only 1% of GSE-1. The remaining 83% were polyphenols, with about 75% attributed to tannins. The remaining polyphenols accounted for about 8% of the total CAD signal. That 8% contributed 41 constituents and were attributed to be monomers (e.g., catechin), OPC (i.e., dimers to pentamers), or lignans (Table 1, Table S1).

### Confirmation of tannin content

Based on literature (Peng et al., 2001), the broad peaks observed in the chromatograms in Figure 1 were likely tannins, but further analysis was performed to confirm the identification. Following precedents from literature, two tannin removal methods were performed (Peng et al., 2001). The first involved mixing the GSE-1 with poly(vinylpolypyrrolidone) (PVPP), which strongly binds polyphenols. After mixing and preparing for UHPLC-CAD, the broad chromatographic peak was nearly absent in the treated sample (Figure S7—green). However, a majority of the other constituents were also polyphenols and were also removed. In a separate set of experiments, the extract was passed through a 3,000-molecular weight cut-off (MWCO) filter, which removed constituents with a molecular weight >3,000 Da (Figure S7—red). These experiments indicated that the broad peak consisted of large (>3,000 Da) polyphenolic species. Since tannins are a mixture of polymers, an increasing series (3,000, 10,000, 30,000, 50,000, 100,000) of MWCO filters were applied to determine the size distributions of the polymer mixture (Figure S8). After applying the MWCO filters of 3,000, 10,000, 30,000, 50,000, and 100,000, the GSE lost 87, 82, 75, 65, and 50%, respectively, of its overall CAD signals between 10 and 50 min (Figure S8). These data demonstrate that there is a large portion of tannins with high molecular weight (>100,000) and that the tannins are distributed across a wide mass range (Hümmer and Schreier, 2008).

To spectrally confirm the identification of the tannins, the retentate of a 30,000-MWCO was repeatedly washed, then reconstituted in CH_3_OH and subjected to ESI-QTOF analysis. The flow injection ESI-QTOF data, while not exactly clear, displayed a spectrum indicative of a broad range of high molecular weight species (Figure S9). Additionally, increasing collision energies gave fragment ions that confirmed the composition of these high molecular weight species. These fragment ions included catechin, varying degrees of polymerized catechin (and their galloylated analogs), and sugar moieties (Figure S9). Thus, the broad chromatographic peak in the GSE-1 CAD chromatogram was a large molecular weight species made up of polymerized catechin, also defined as tannins. To confirm that the quantitation of the tannins was correctly accounted for by the original standards, the retentate from the 30,000-MWCO filter experiment was also used as a standard. A calibration curve was generated to calculate a response factor (Figure S10). The response factor from this curve closely matched the response factor that was originally generated by the catechin and proanthocyanidin standards. Thus, the amount of tannins were accurately represented in the original analysis and are about 75% of the GSE.

### Quantitation and characterization by GC-FID and GC-HRMS

One deficiency of the UHPLC-UV-CAD-HRMS system is its inability to accurately analyze volatile compounds. Liquid chromatography can have issues handling certain volatile compounds and the CAD has inconsistencies with compounds that have a boiling point < 400°C. Therefore, to quantitate the contribution of mass from the volatile compounds, GC-FID was used. The GSE-1 was dissolved fully in CH_3_OH and injected into the GC-FID system. Hydrocarbon standards (C6, C7, C9, and C10-C25) were used to quantify the volatiles in the GSE-1. Based on GC-FID data, a calculated mass balance of 2.6% was achieved in this experiment. This was added to the already existing 91% detected by UHPLC-CAD to account for 93.6% of the mass overall. Within the 2.6% of volatile constituents in the GSE, there were 8 individual constituents with a mass above the toxicological threshold of interest. These 8 constituents account for about 1.4% of the GSE. The remainder (1.2%) of the 2.6% is made up of smaller peaks that do not exceed the threshold individually.

To identify the contributing volatile compounds, the GSE was analyzed via GC-HRMS. The eight peaks that had a mass above the toxicological threshold were characterized using chemical ionization (CI) and electron ionization (EI) MS. CI-HRMS data identified the protonated molecules (identification of molecular formula) and the fragmentation by EI was searched against the NIST 14 database for the identification (Table 2, Table S2). The Kovats retention indexes were calculated to correlate the peaks from the GC-FID to those on the GC-HRMS. For the eight constituents, five were matched with reference standards, three were partial matches with only a molecular formula assigned. The partial matches are believed to be related to substituted phenolic constituents based on the double bond equivalents and fragmentation. The eight constituents were identified as glycerol, dihydroxyacetone, and substituted phenols (e.g., catechol, 4-methylbenzenediol, etc.).

**Table 2:** Proposed identifications by accurate mass GC-HRMS of the components producing significant peaks in the GC-FID analysis of GSE-1.

**FID Peak**	**Kovats retention index**	**Accurate *m/z* (ppm)**	**Proposed identification**	**Molecular formula**	**Amt. μg per 210 mg dose**	**Confidence (A-C)**	**Accurate mass chemical ionization matches formula**	**Accurate mass electron ionization matches formula**	**Kovats retention index matches reference**
1	732	87.0441 (−0.006)	*Unidentified*	C_4_H_6_O_2_	170	Partial	✓		
2	894	91.0390 (0.192)	Dihydroxyacetone	C_3_H_6_O_3_	710	Reference	✓	✓	✓
3	966	91.0390 (0.039)	Glycerol	C_3_H_8_O_3_	980	Reference	✓	✓	✓
4	1188	111.0441 (0.216)	Catechol	C_6_H_6_O_2_	850	Reference	✓	✓	✓
5	1284	125.0597 (−0.288)	4-methyl catechol	C_7_H_8_O_2_	190	Reference	✓	✓	✓
6	1362	127.0390 (−0.084)	1,2,3-benzenetriol	C_6_H_6_O_3_	770	Reference	✓	✓	✓
7	1593	153.0543 (−1.768)	*Unidentified*	C_8_H_8_O_3_	620	Partial	✓		
8	1801	165.0544 (−1.579)	*Unidentified*	C_9_H_8_O_3_	230	Partial	✓		

*^A^Confirmed constituent was not 1,3-benzenediol nor 1,4-benzenediol based on retention time and EI-HRMS using standards*.

*^B^Confirmed constituent was not 3-methyl catechol nor orcinol based on retention time and EI-HRMS using standards*.

*^C^Confirmed constituent was not 1,3,5-Benzenetriol nor 1,2,4-Benzenetriol based on retention time and EI-HRMS using standards*.

### Outcome of the *in silico* safety assessment

A series of toxicological studies underpin the safety of proanthocyanidin-rich extracts from grape seeds but the available data indicate a lack of investigation into developmental parameters (Yamakoshi et al., 2002). There has been no indication of adverse reproductive or developmental effects in humans from dietary exposure to GSE (nor some of the individual components that have been tested), but due to the lack of developmental data, focus was given to constituents with a resulting exposure above Cramer Class III in the TTC approach (European Food Safety and World Health, 2016). The application of TTC in an *in silico* approach to assess safety of botanical dietary supplement ingredients has been discussed previously but in brief is based, in part, on targeting a lower limit of detection for constituent characterization that enables a TTC approach (Little et al., 2017). This allows for a worst-case risk assessment to be performed for specific chemical entities. Cramer Classes I and III are considered sufficiently protective for adverse effects on reproduction or development. Thus, efforts were made to rule out known classes of developmental toxicants (e.g., through careful review of the literature) and the most conservative value of 90 μg per day (Cramer Class III) can be set as a limit of detection for the ensuing constituent characterization.

The decision tree approach described previously by Little et al. (2017) was applied to the GSE constituent data. Namely, to first determine whether constituents with known structures are commonly consumed in the diet and, if so, whether the dietary supplement exposure is comparable to food intake. For constituents above food intakes or those not commonly consumed as food, published safety data is then reviewed to determine if the data are sufficient to establish a suitable margin of safety (MoS). Depending on the outcome of these evaluations an *in silico* assessment process can then be applied, leveraging structure activity relationships (SAR) to fill data gaps or identify toxicity alerts in the absence of information, TTC are applied for specific chemical entities that fit within its constraints. If following this process safety endpoint gaps still remain, then the placing of follow up toxicity studies must be considered, for which the understanding of the composition is required for study design.

The UHPLC-UV-CAD-HRMS and GC-FID & GC-HRMS analysis of GSE showed that tannins are the key constituent class, comprising about 75% of the total composition. Other constituents present include flavonoids (~7%), lignans (<1%), and food components (16%), such as salts and sugars.

A broad range of high molecular weight tannins were observed, and in terms of amount this tannin peak approximated 160,000 μg per 210 mg GSE dose. Tannin-rich foods and beverages include berries, cocoa/chocolate, green tea, black tea, red wine, and coffee and Western diet exposures range from several tens to several hundreds of milligrams per day (Santos-Buelga and Scalbert, 2000; Prior and Gu, 2005; Serrano et al., 2009). Thus, the use of GSE containing the tannin content represented by the majority of extracts examined in this study is comparable to the diet.

With regards to the flavonoids present, these approximate 15,000 μg per 210 mg GSE dose include non-galloylated and galloylated flavonoids with varying levels of oligomerization (dimer to pentamer). For the purpose of this assessment, the data on the flavanoids were obtained from the Phenol-Explorer Database (Neveu et al., 2010; Rothwell et al., 2012, 2013) and exposure estimates made using daily ingestion of foodstuffs taken from the Food Commodity Intake Database (2017b). This work demonstrated that these constituents are present in a number of commonly consumed foods such as black/green tea, cocoa, and a variety of fruits and vegetables, with dietary exposures from several milligrams to several tens of milligrams. Thus, the use of GSE provides a daily exposure to flavonoids that is comparable to the diet.

The lignans present in the GSE approximate 550 μg per 210 mg GSE dose and are also found in grains and seeds such as barley, buckwheat, oat, rye, wheat, flaxseed, sesame seed. Although at lower levels they are also found in some common fruits and vegetables and maple syrup (Peterson et al., 2010; Li and Seeram, 2011). Lignan intake does not usually exceed 1 mg per day in most Western populations but estimates of lignan intakes can approach about 1,600 μg per day (Milder et al., 2005; Peterson et al., 2010). Thus, the use of GSE provides a daily exposure to lignans that is comparable to the diet.

Following the characterization work there were a small number of peaks where MS/MS could not assign a structure. In addition, one flavonoid constituent was identified that was not found in food (Embigenin, CAS# 21089-34-9) based on currently available data sources. It is classified as a tentative structure and present at 35 μg per 210 mg GSE dose. Total exposure to these components approximated 150 μg, with individual components ranging from 4.5 to 60 μg. Since each of these unknowns and the one flavonoid fall below the TTC value assigned to the exercise (i.e., 90 μg per day) no further work was deemed necessary.

In conclusion, the qualitative and quantitative characterization of the constituents present in the GSE demonstrates a high similarity to components of commonly consumed food. Hence, the safety data gap for GSE at doses up to 210 mg per day can be addressed by benchmarking constituent data to commonly ingested food components, with comparable dietary exposures.

### Comparison of detectors

**Figure 4:**
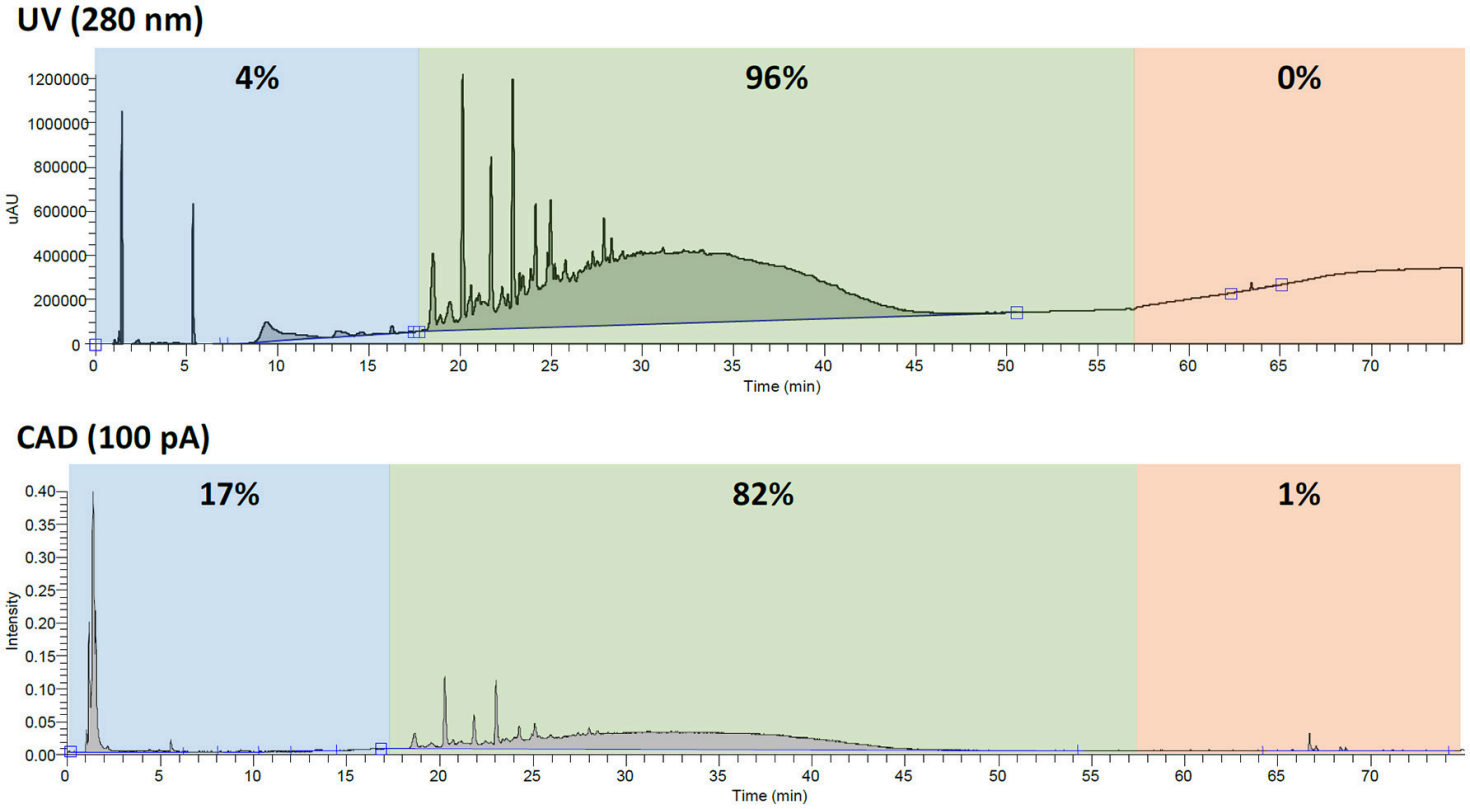
The percentages (calculated by area under the curve) of polar compounds (blue), polyphenols (green), and nonpolar compounds (red) when analyzed by UV (280 nm) and CAD (100 pA). The three time zones were selected to loosely represent the different types of compounds.

When quantifying constituents of complex mixtures, it is important to make sure you are accounting for all the constituents of a sample. This is especially true when working with dietary supplements since it is required to report nutrient content such as fat, sugar, cholesterol, etc. (2017a). Since botanicals and other natural products will inherently contain sugars, amino acids, fatty acids, and other natural components, a detector that can observe the various analytes is beneficial. For instance, while mass spectrometry is a highly sensitive detector, its response varies greatly due to the ionization efficiency of the analyte. Additionally, while UV has been the industrial standard due to its availability and affordability, it requires that the constituent have a chromophore. Both of these detectors suffer when looking at such complex mixtures as botanicals. In the case of GSE, proanthocyanidin content can easily be overestimated due to the underrepresentation of polar (e.g., salts, sugars, amino acids, etc.) or nonpolar compounds (e.g., sterols, fatty acids, etc.) by UV. For example, using UV, the polyphenolic content in GSE-1 would be estimated at 96% with only 4% polar compounds and almost no nonpolar compounds accounted for. Looking at the same sample by CAD, these numbers greatly shift to reveal that about 17% of the sample was polar compounds and about 1% was nonpolar. The CAD determined 82% polyphenols was a significant difference from the 96% that was determined by UV (Figure 4), not taking volatiles into account.

### Comparison of GSE suppliers

To better capture an overview of the GSE suppler market, GSEs were obtained from three additional suppliers (GSE-2 through GSE-4) with two different lots from GSE-3 and GSE-4 (a & b). Note that these are suppliers of grape seed extracts and not commercialized grape seed extract dietary supplements. These GSE samples were all analyzed using the UHPLC-UV-CAD-HRMS system, and a comparison of their proanthocyanidin content was made (Figure S11). The percentages of total polyphenols, tannins (DP ≥ 6), non-tannin polyphenols [(epi)catechin, OPC, etc.], gallic acid, polar compounds, and nonpolar compounds were calculated (Table 3). The results highlighted a few important details: (1) the GSE from various suppliers were generally similar, (2) they all were predominately tannins, and (3) using a universal, unbiased detector was important for a true representation of the botanical's contents.

**Table 3:** The percentages of compound classes for the authenticated GSE voucher, GSE-1, and a variety of other GSE suppliers (GSE-2 to 4).

	**% Polyphenols**		**% Tannins**		**% Non-tannin Polyphenols**		**% Gallic Acid**		**% Polar**		**% Nonpolar**	
	**UV**	**CAD**	**UV**	**CAD**	**UV**	**CAD**	**UV**	**CAD**	**UV**	**CAD**	**UV**	**CAD**
Voucher	98	56	94	54	4	2	1	0	0	38	0	6
GSE-1	99	82	87	72	13	10	0	0	0	17	0	1
GSE-2	87	92	74	81	13	11	10	3	3	5	0	0
GSE-3a^*^	98	93	92	87	6	6	1	0	0	6	0	0
GSE-3b^*^	98	93	89	83	9	9	2	0	0	8	0	0
GSE-4a^*^	100	89	91	81	9	8	0	0	0	11	0	0
GSE-4b^*^	100	92	90	83	9	9	0	0	0	7	0	0

*Percentages are the average of three separately prepared 10 mg/mL samples dissolved in 70% ACN, 70% MeOH, or 50% MeOH in H_2_O. The %RSD was below 10% for the three dissolutions of each sample*.

* *Annotations of “a” or “b” indicate that two different lots/batches were analyzed from the same supplier*.

The general composition of the GSEs were largely similar with a majority of the extracts being tannins (Table 3). GSE-1 had the largest percentages of polar (17%) and nonpolar (1%) compounds. The other GSEs did not have nearly as much polar/nonpolar compounds in the sample, which was potentially due to a clean-up step in the manufacturer's extraction process. Interestingly, GSE-2 stood out with a higher percentage of gallic acid (3%) compared to the other GSEs (<0.5%). This was even further exaggerated when looking at the UV data, where the gallic acid content reached 10% and the others are all ≤ 2%. While the reason for this is unknown, this variation between extracts is not unprecedented (Villani et al., 2015). This has been observed before in literature, as a publication that outlined GSE botanical content had 3 authentic GSE reference standards, and one of them also had a higher gallic acid content (Villani et al., 2015). For GSE-3 and GSE-4, two separate lots were analyzed for each. For GSE-3, there were some differences in the percentages for the tannin and non-tannin polyphenols, but overall were similar each other and to the other GSEs. These types of differences are to be expected between lots and even suppliers as there are many variables to account for (e.g., dried extract homogeneity, harvesting time of year and location, etc.). GSE-4a and GSE-4b were largely similar with one having a higher polar compound percentage. Once again, these were considered negligible differences (Table 3) and the GSEs were overall similar in OPC content.

The authentic grape seed material (voucher) that was extracted in-house, was also analyzed for comparison. This sample presumably has much less processing than the industrial processes used by suppliers. When analyzed by the CAD, the proanthocyanidin percentages were significantly lower due to the abundance of polar compounds (e.g., sugars, amino acids, etc.). However, when analyzed by UV, the authentic GSE did not look much different from the supplier GSEs, even though based on the CAD, it was almost 40% polar type compounds. This comparison highlights the differences between using an unbiased, universal detector like the CAD as opposed to UV.

It is worth emphasizing that this multi-detector platform afforded the ability to obtain different chromatograms that were often complimentary (Table 4). Complex materials, such as these botanicals, analyzed by CAD, UV, positive and negative ion MS, with suitable chromatographic introduction, were compared using the four complimentary data sets. For instance, although many analytes were detected by both ionization modes, some analytes were observed only by either positive or negative ion ESI-MS. Furthermore, some large molecular weight species were not detectable in either ionization mode (e.g., tannins). The UV detector provided sensitive detection of analytes but required that the constituent contained a chromophore. UV also added another orthogonal data set (UV spectra) that aided the identification and comparison of samples. While the CAD doesn't provide spectral data, it did detect constituents where MS and UV were deficient. Analytes that were not observed well by ESI-MS (e.g., tannins) nor by UV (e.g., sugars and fatty acids), were readily detected by CAD.

**Table 4:** Comparison of the UV, CAD, and MS detectors to highlight the importance of using a multi-detector system for constituent identification.

	**CAD**	**UV**	**ESI-MS**
Detection	Universal detection for constituents with a boiling point >400°C	Requires the constituent to have a chromophore and is affected by conjugation	Requires the constituent to ionize and is affected by the ionization efficiency
Quantitation	Any standard	Requires a standard for each class of compounds in the sample	Requires a standard for each compound in the sample
Spectral data for characterization	✘	✔	✔
**CONSTITUENT DETECTION CAPABILITIES/LIMITATIONS**			
Polyphenols	✔	✔	✔
Sugars	✔	✘	✔
Fatty acids	✔	✘	✔
Tannins	✔	✔	✘

In conclusion, an evaluation of a botanical for use in a dietary supplement should account for the chemical complexities inherent to natural products. In this analysis of grape seed extracts, the use of a universal detector, such as the CAD, provided unbiased quantitative information and the incorporation of GC-FID and GC-HRMS to the traditional UHPLC-MS analysis (VanderMolen et al., 2017) allowed for a comprehensive qualitative investigation of the sample. The summation of these techniques provided important information for the toxicological safety assessment that helped obviate the need for classical *in vitro* and *in vivo* safety studies (VanderMolen et al., 2017). Finally, by comparison of various GSE materials, a better understanding of the GSE botanical market was gained.

### Associated content

#### Supporting information

Chromatograms comparing GSE-1 to authentic grape seed, peanut skin, and pine bark extracts (Figures S1–S4), the elucidation of a new compound (Figure S5), the selected CAD peaks that were investigated (Figure S6), the overlaid chromatograms for tannin removal (Figures S7, S8), the QTOF spectra of the tannins (Figure S9), the calibration curves for the standards and tannins (Figure S10), the CAD chromatograms comparing various GSE suppliers (Figure S11), and the tabulated spectral data (Tables S1, S2) for each constituent are contained in the Supplemental Information.

## Author contributions

VS conducted the sample preparation, analyses, data analyses, and primary manuscript authorship. CM led the *in silico* safety assessment and contributed to the writing of the manuscript. TB was the PI for the project and contributed to the experimental design and interpretation, as well as editing of the manuscript.

### Conflict of interest statement

The authors declare that the research was conducted in the absence of any commercial or financial relationships that could be construed as a potential conflict of interest.
