# Supplementary material for: Multi-Detector Characterization of Grape Seed Extract to Enable in silico Safety Assessment
Source: Front Chem. 2018 Aug 14;6:334. doi: 10.3389/fchem.2018.00334 (PMC6102626; doi:10.3389/fchem.2018.00334)
Supplement: Supplementary file 1 [file Data_Sheet_1.pdf]

Multi-detector Characterization of Grape Seed Extract to Enable *In Silico* Safety Assessment

Vincent P. Sica,[1] Catherine Mahony,[2] and Timothy R. Baker[1]

[1]Research and Development, Corporate Functions Analytical, The Procter & Gamble Company, Mason Business Center, 8700 Mason-Montgomery Rd, Mason, OH 45040, United States

[2]Central Product Safety, The Procter & Gamble Company Technical Centres Ltd, Egham, United Kingdom

Figure S1. The stacked negative mode UHPLC-HRMS chromatograms of the GSE-1 extract, the extracted authentic grape seed (*Vitis vinifera)* material, the extracted authentic peanut skin (*Arachis* sp.) material (green trace), and the extracted authentic pine skin (*Piunus pinaster*) material (blue trace). The GSE-1 and authentic grape seed extracts were in agreement. There were no indications of adulteration in the GSE-1 extract when compared to the peanut skin and pine bark traces.

Figure S2. The stacked UHPLC-UV chromatograms of the GSE-1 extract, the extracted authentic grape seed (*Vitis vinifera)* material, the extracted authentic peanut skin (*Arachis* sp.) material (green trace), and the extracted authentic pine skin (*Piunus pinaster*) material (blue trace). The GSE-1 and authentic grape seed extracts were in agreement. There were no indications of adulteration in the GSE-1 extract when compared to the peanut skin and pine bark traces.

Figure S3. The narrow mass chromatograms for *m/z* 575.1189 (±5ppm) for GSE-1, authentic GSE, authentic peanut skins extract, authentic pine bark extract, and standards of proanthocyanidin A1 and A2 showed that GSE-1 and the authentic grape seed extract did not contain the key indicators of peanut skin and/or pine bark adulteration (proanthocyanidin A-type compounds).

Figure S4. (A) Overlay of the GSE-1 (black) and authentic GSE (red) CAD chromatograms from 18-30 minutes, highlighting an outlier in the GSE-1 sample. All other CAD peaks from the GSE-1 are accounted for in the authentic GSE. (B) The negative mode MS/MS spectrum at 23.3 min for the outlier in GSE-1 matched the MS/MS pattern for (C) 1-(3',4'-dihydroxyphenyl)-3-(2",4",6"-trihydroxyophenyl)propan-2-ol in literature.

Figure S5. A comparison of (A) the proposed structure for a degraded proanthocyanidin dimer based on MS/MS interpretation and (B) an isomer from literature with its reported MS/MS interpretation. Compared to Proanthocyanidin B1, the propose structure was missing a bond in the B'-ring, while the reported isomer was missing the bond in the B-ring. (C) The MS/MS spectrum of the proposed structure displayed similar types of fragmentation to the isomer in literature, but the differences in structure resulted in different observed masses. NOTE: RDA indicates where the fragment was generated via Retro Diels-Alder.

Figure S6. The CAD chromatogram of GSE-1 with each CAD peak labeled. The chromatogram was divided into three sections (0-17, 17-46, and 46-75 min) for clarity. The 39th peak is the broad peak ranging from about 18-50 min. The small peaks that are not labeled were below the threshold of toxicological concern.

Figure S7. Overlaid UHPLC-CAD chromatograms of the GSE-1 (black trace), GSE-1 treated with poly(vinylpolypyrrolidone) (PVPP) (green trace), GSE-1 filtered through a molecular weight cut-off

(MWCO) filter at 3000 Da (red trace), and the diluent blank (blue trace) showed the removal of the large chromatographic hump and supported its identity as tannins.

Figure S8. The overlaid chromatograms of GSE-1 filtered through a molecular weight cut-off (MWCO) filters at 3,000 Da (black traces), 10,000 Da (red traces), 30,000 Da (green traces), 50,000 Da (blue traces), and 100,000 Da (yellow traces). The top chromatograms represent the filtrate (passed through filter) and the bottom chromatograms represent the retentate (didn't pass through filter).

Figure S9. The retentate from the GSE after passing through a 30,000 MWCO filter was subjected to ESI-QTOF. With a collision energy (CE) of 10V, signals indicating high molecular weight tannin species was detected (top). As the CE was increased, fragments relating to sugars (*m/z* 162), catechin (*m/z* 288), and catechin-polymers (*m/z* 576, 864, etc.) were detected, further supporting the evidence for tannins.

Figure S10. The calibration curves generated by area under the curve (AUC) from the CAD chromatograms for the four standards (top) compared to the isolated tannins (bottom). To the right of each are the averages all the calculated response factors (AUC/concentration) and their respective standard deviations.

Figure S11. (A) The CAD and (B) UV (280 nm) chromatographic profiles for the authenticated GSE voucher and the various extracts from different suppliers.

Table S1. Proposed identifications of components producing CAD peaks in the UHPLC-UV-CAD-HRMS/MS analysis of grape seed (*Vitis vinifera*) extract for GSE-1.

Table S2. Proposed identifications of components producing FID peaks in the GC-FID and GC-HRMS analysis of grape seed (*Vitis vinifera*) extract for GSE-1.

Supplemental Information

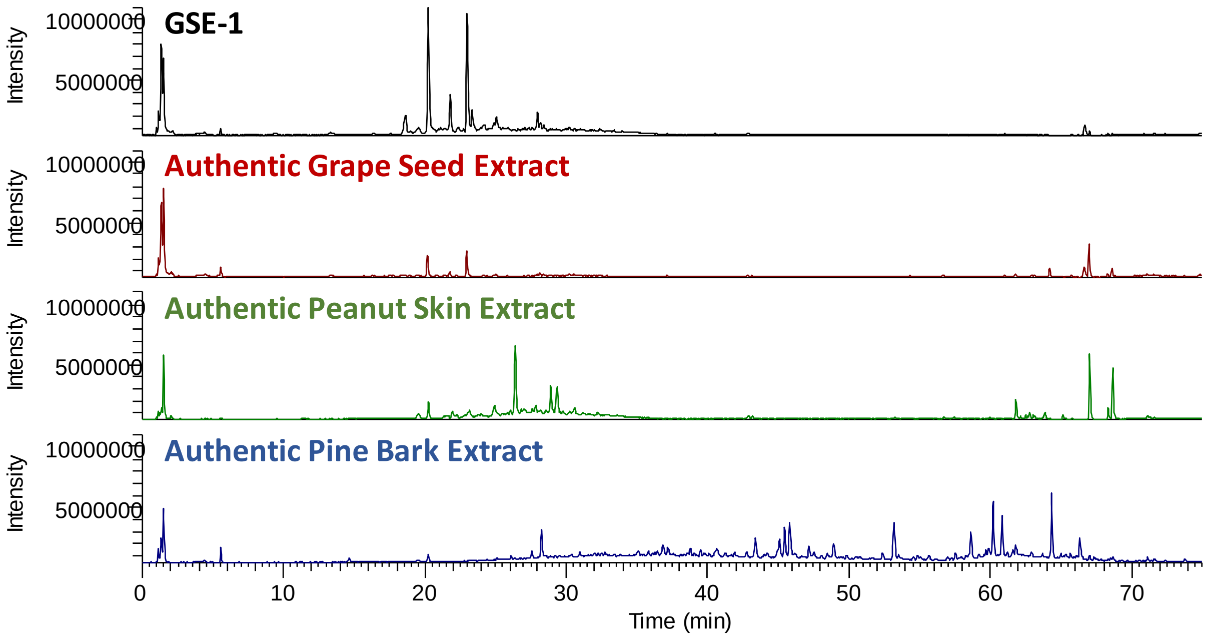

Figure S1. The stacked negative mode UHPLC-HRMS chromatograms of the GSE-1 extract, the extracted authentic grape seed (*Vitis vinifera)* material, the extracted authentic peanut skin (*Arachis* sp.) material (green trace), and the extracted authentic pine skin (*Piunus pinaster*) material (blue trace). The GSE-1 and authentic grape seed extracts were in agreement. There were no indications of adulteration in the GSE-1 extract when compared to the peanut skin and pine bark traces.

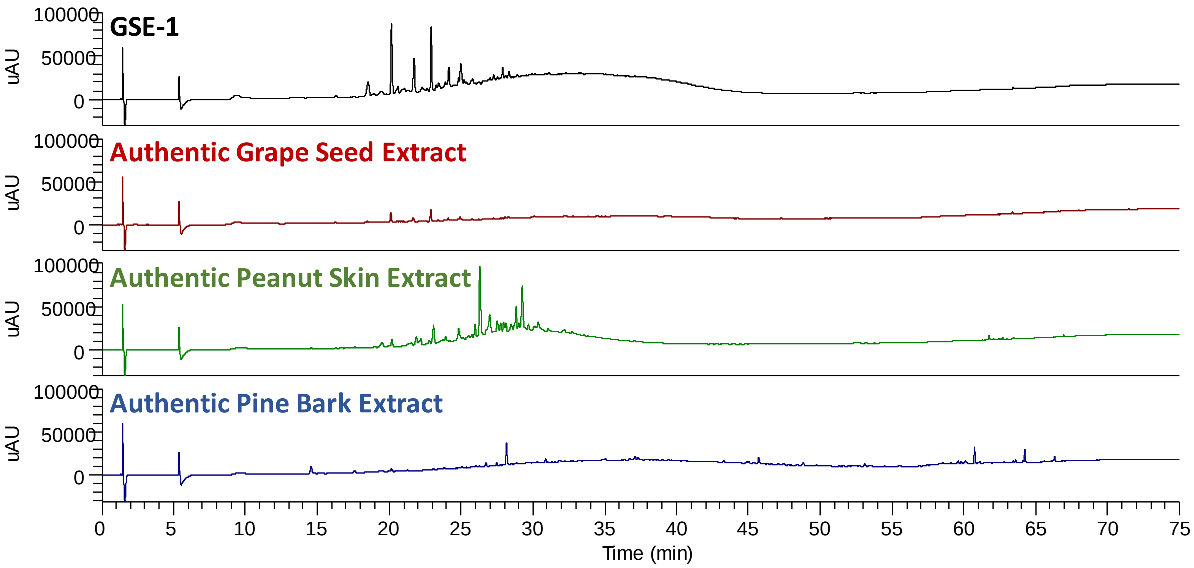

Figure S2. The stacked UHPLC-UV chromatograms of the GSE-1 extract, the extracted authentic grape seed (*Vitis vinifera)* material, the extracted authentic peanut skin (*Arachis* sp.) material (green trace), and the extracted authentic pine skin (*Piunus pinaster*) material (blue trace). The GSE-1 and authentic grape seed extracts were in agreement. There were no indications of adulteration in the GSE-1 extract when compared to the peanut skin and pine bark traces.

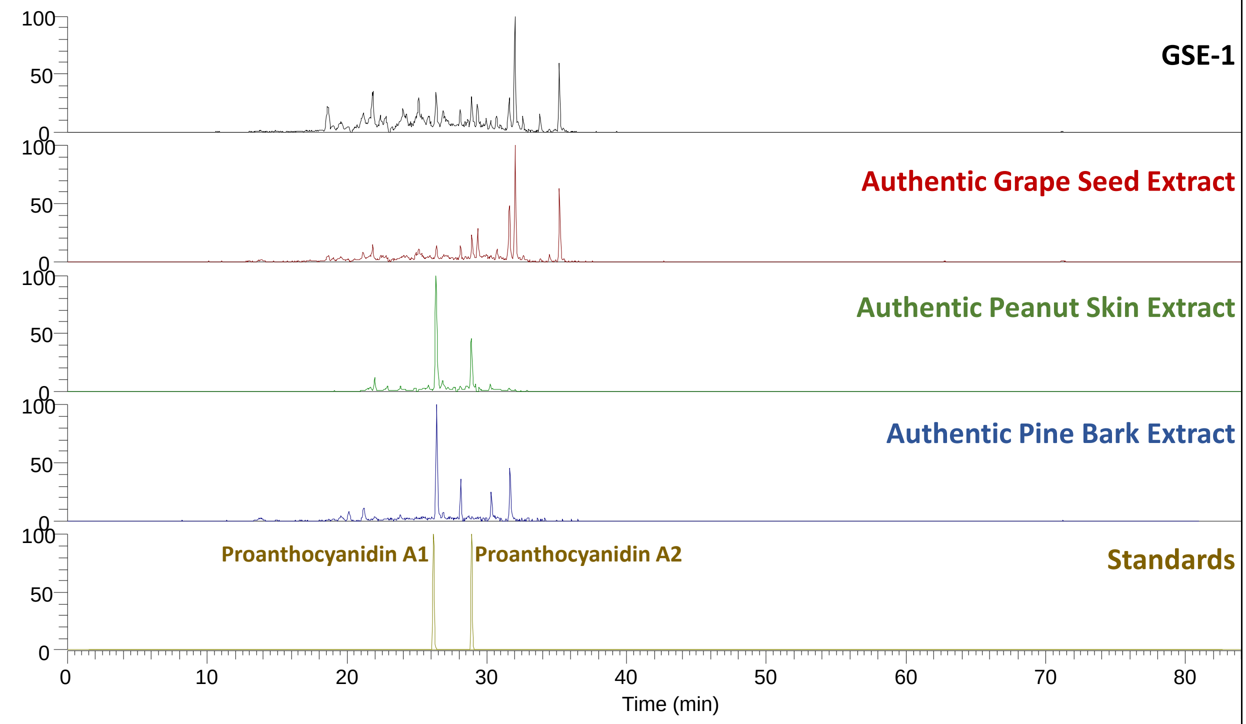

Figure S3. The narrow mass chromatograms for *m/z* 575.1189 (±5ppm) for GSE-1, authentic GSE, authentic peanut skins extract, authentic pine bark extract, and standards of proanthocyanidin A1 and A2 showed that GSE-1 and the authentic grape seed extract did not contain the key indicators of peanut skin and/or pine bark adulteration (proanthocyanidin A-type compounds).

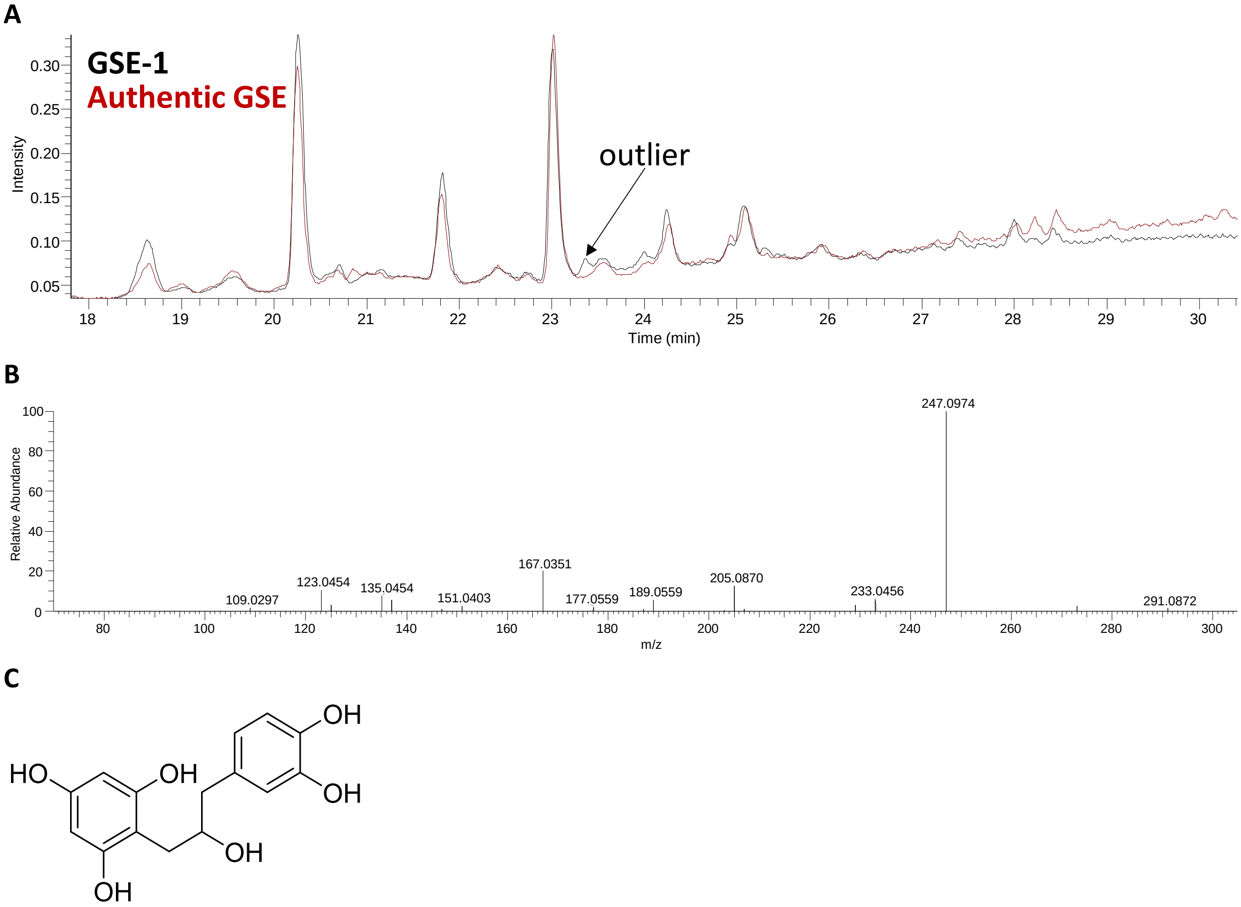

Figure S4. (A) Overlay of the GSE-1 (black) and authentic GSE (red) CAD chromatograms from 18-30 minutes, highlighting an outlier in the GSE-1 sample. All other CAD peaks from the GSE-1 are accounted for in the authentic GSE. (B) The negative mode MS/MS spectrum at 23.3 min for the outlier in GSE-1 matched the MS/MS pattern for (C) 1-(3',4'-dihydroxyphenyl)-3-(2",4",6"-trihydroxyophenyl)propan-2-ol in literature.[19]

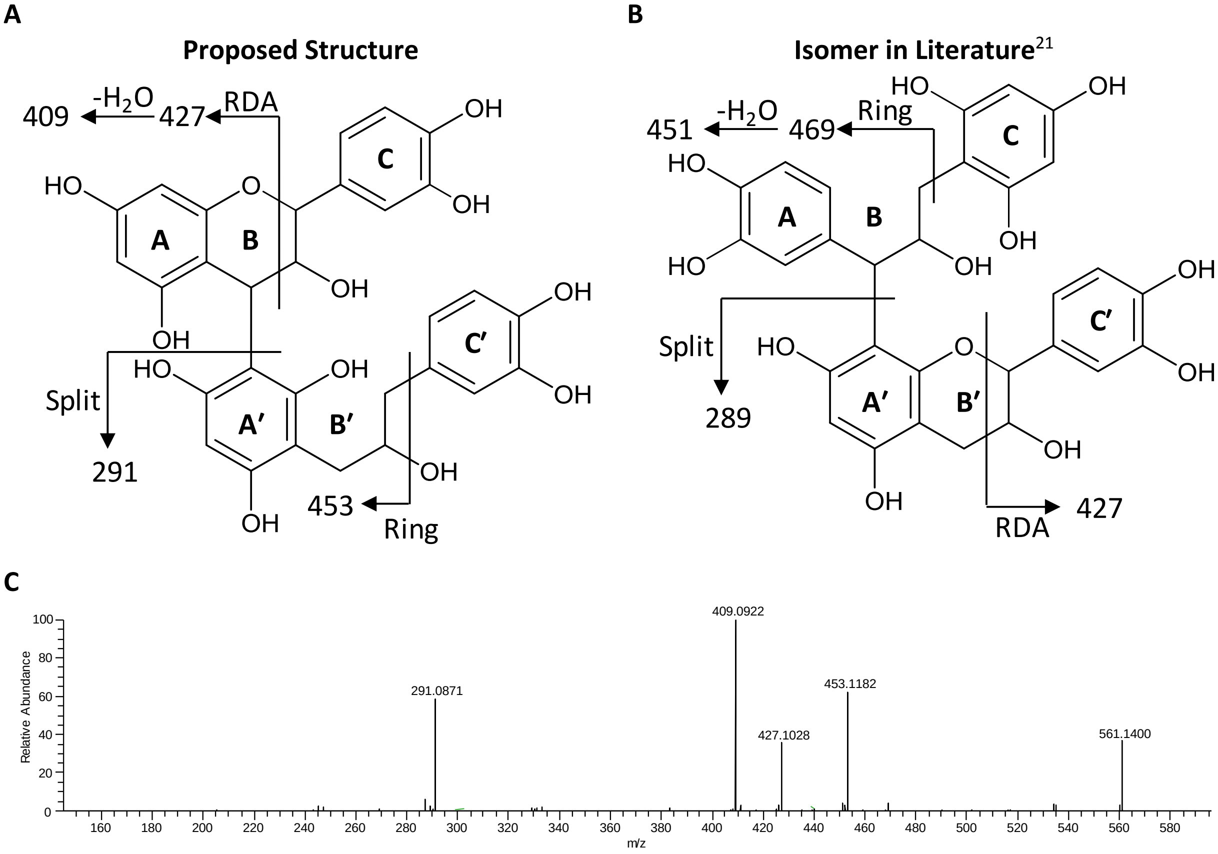

Figure S5. A comparison of (A) the proposed structure for a degraded proanthocyanidin dimer based on MS/MS interpretation and (B) an isomer from literature with its reported MS/MS interpretation.[21] Compared to Proanthocyanidin B1, the propose structure was missing a bond in the B′-ring, while the reported isomer was missing the bond in the B-ring. (C) The MS/MS spectrum of the proposed structure displayed similar types of fragmentation to the isomer in literature,[21] but the differences in structure resulted in different observed masses. NOTE: RDA indicates where the fragment was generated via Retro Diels-Alder.

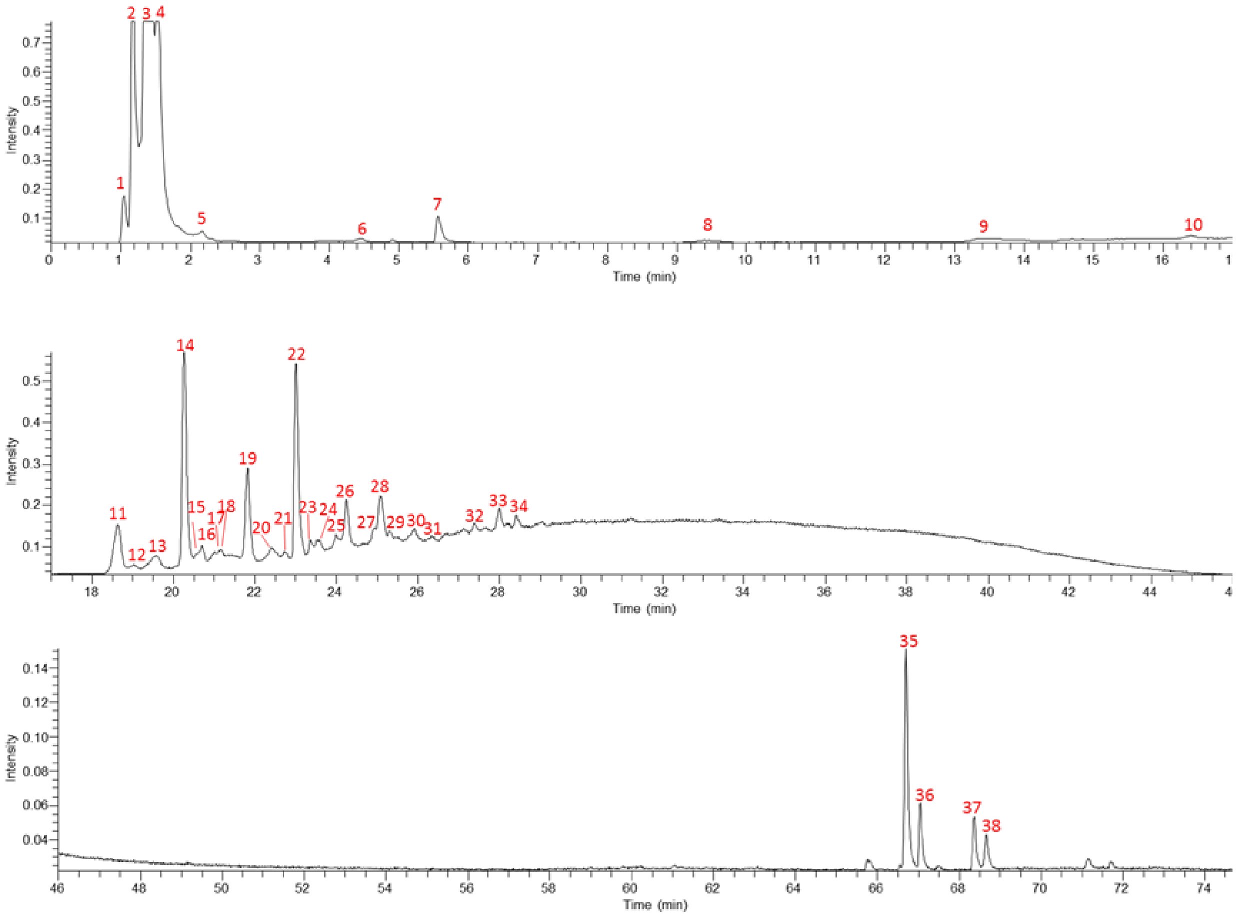

Figure S6. The CAD chromatogram of GSE-1 with each CAD peak labeled. The chromatogram was divided into three sections (0-17, 17-46, and 46-75 min) for clarity. The 39th peak is the broad peak ranging from about 18-50 min. The small peaks that are not labeled were below the threshold of toxicological concern.

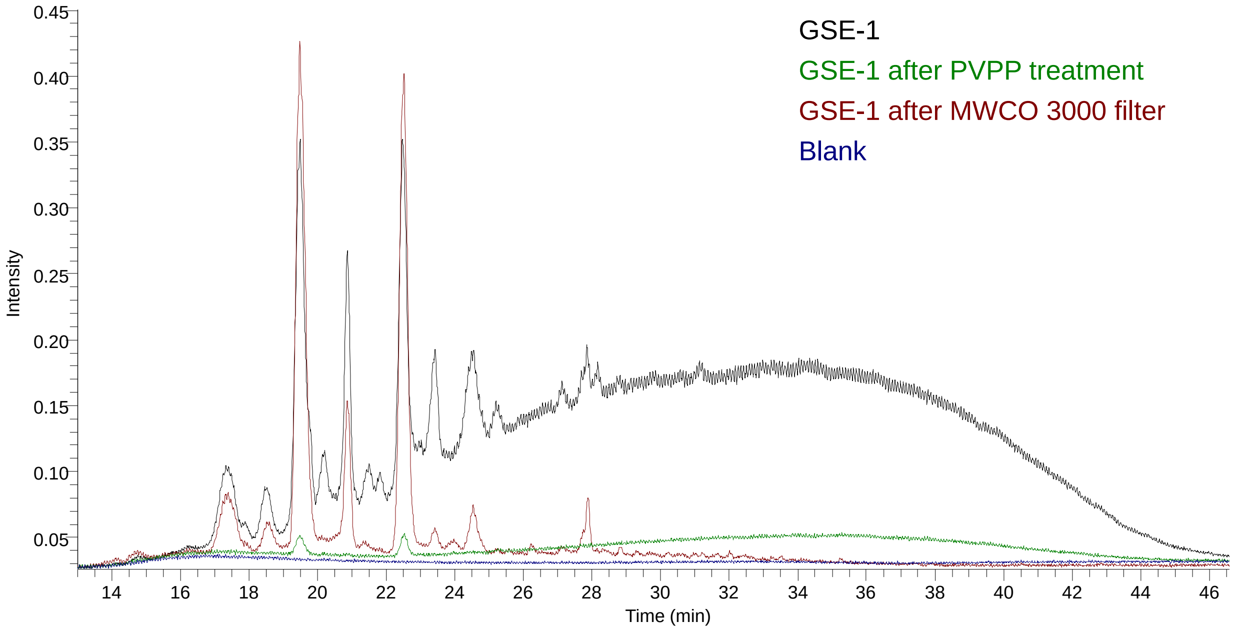

Figure S7. Overlaid UHPLC-CAD chromatograms of the GSE-1 (black trace), GSE-1 treated with poly(vinylpolypyrrolidone) (PVPP) (green trace), GSE-1 filtered through a molecular weight cut-off (MWCO) filter at 3000 Da (red trace), and the diluent blank (blue trace) showed the removal of the large chromatographic hump and supported its identity as tannins.

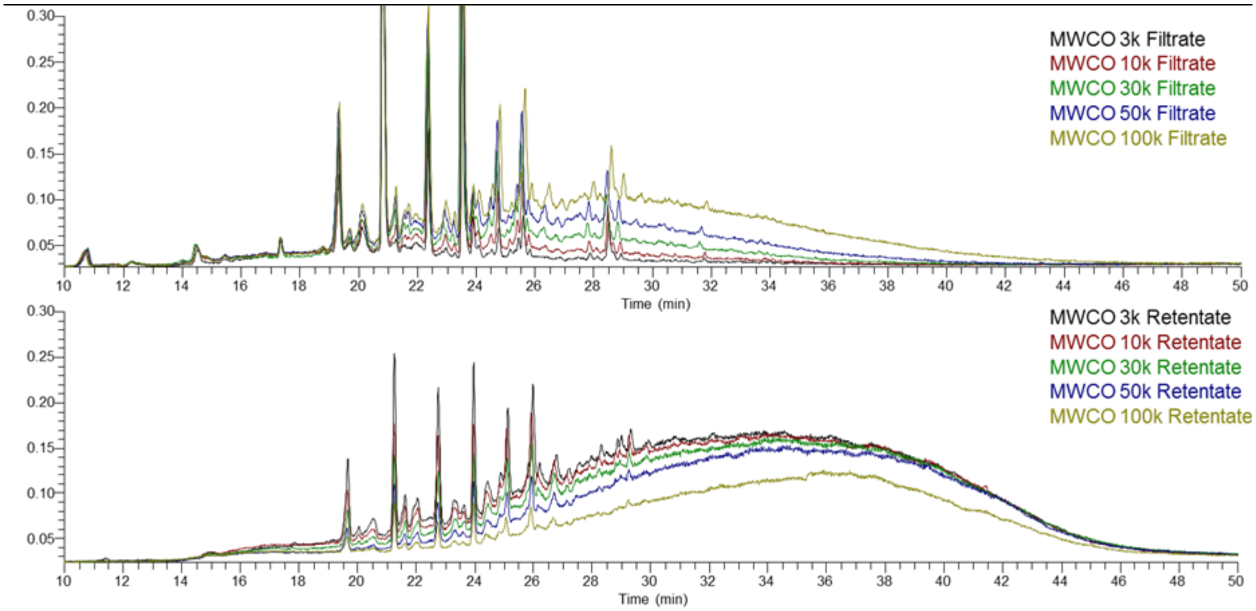

Figure S8. The overlaid chromatograms of GSE-1 filtered through a molecular weight cut-off (MWCO) filters at 3,000 Da (black traces), 10,000 Da (red traces), 30,000 Da (green traces), 50,000 Da (blue traces), and 100,000 Da (yellow traces). The top chromatograms represent the filtrate (passed through filter) and the bottom chromatograms represent the retentate (didn't pass through filter).

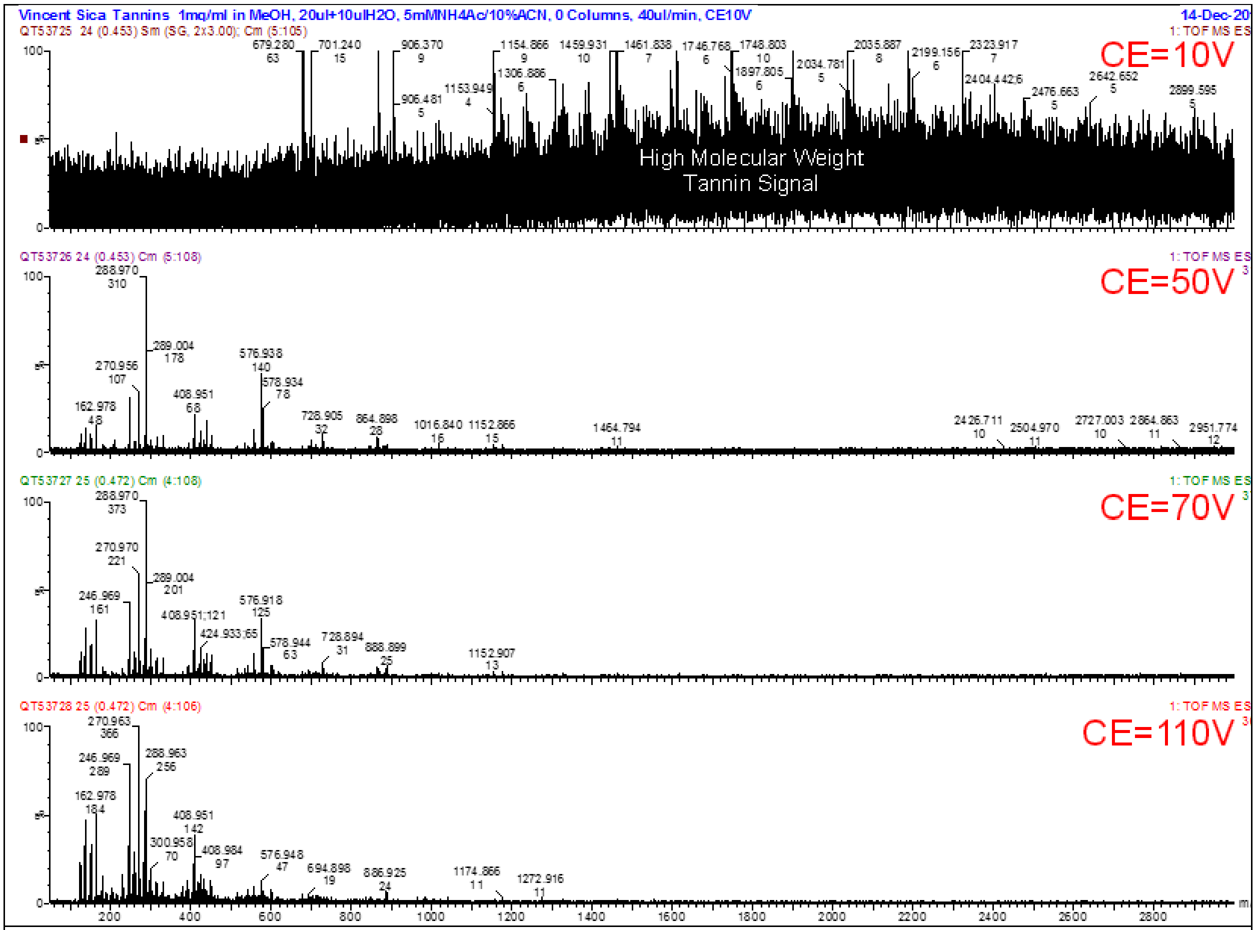

Figure S9. The retentate from the GSE after passing through a 30,000 MWCO filter was subjected to ESI-QTOF. With a collision energy (CE) of 10V, signals indicating high molecular weight tannin species was detected (top). As the CE was increased, fragments relating to sugars (*m/z* 162), catechin (*m/z* 288), and catechin-polymers (*m/z* 576, 864, etc.) were detected, further supporting the evidence for tannins.

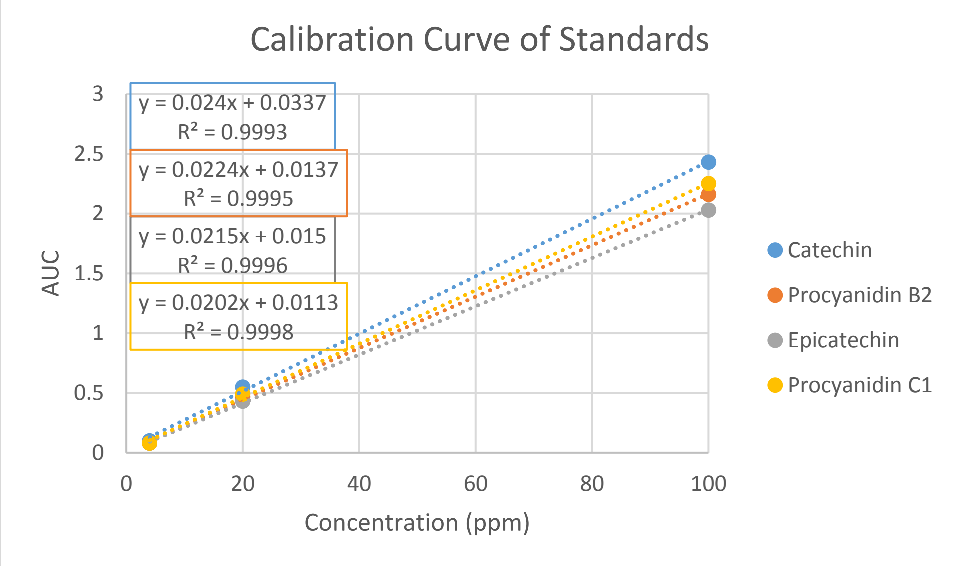

Average Response Factor (RF): 0.0226

Standard Deviation of Response Factor: 0.002

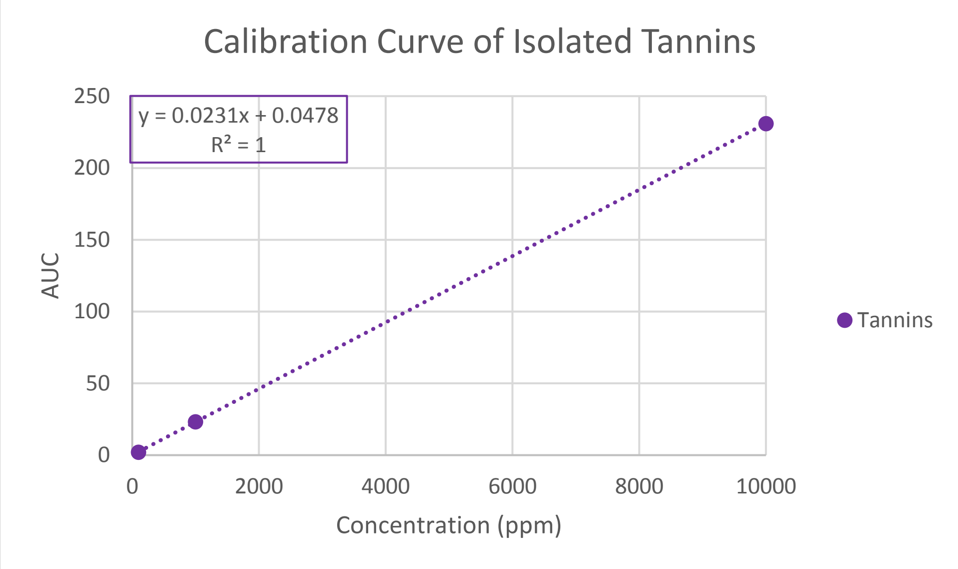

Average Response Factor: 0.0227

Standard Deviation of Response Factor: 0.001

Figure S10. The calibration curves generated by area under the curve (AUC) from the CAD chromatograms for the four standards (top) compared to the isolated tannins (bottom). To the right of each are the averages all the calculated response factors (AUC/concentration) and their respective standard deviations.

A

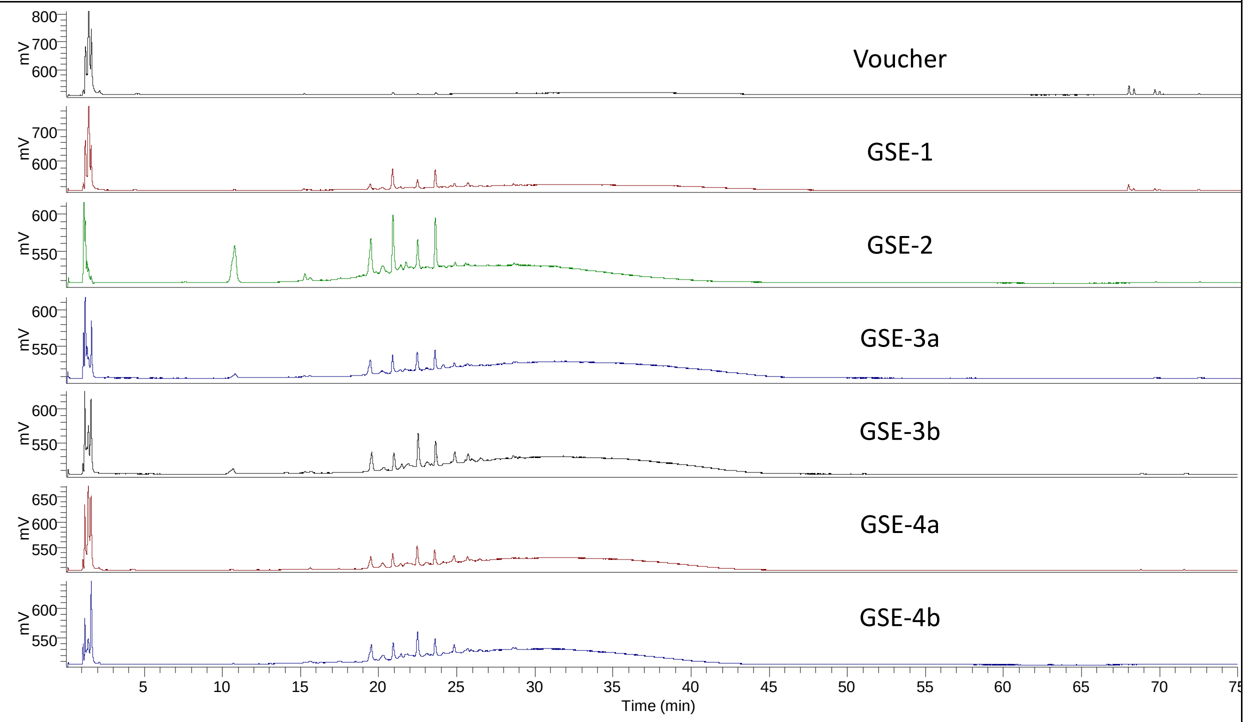

B

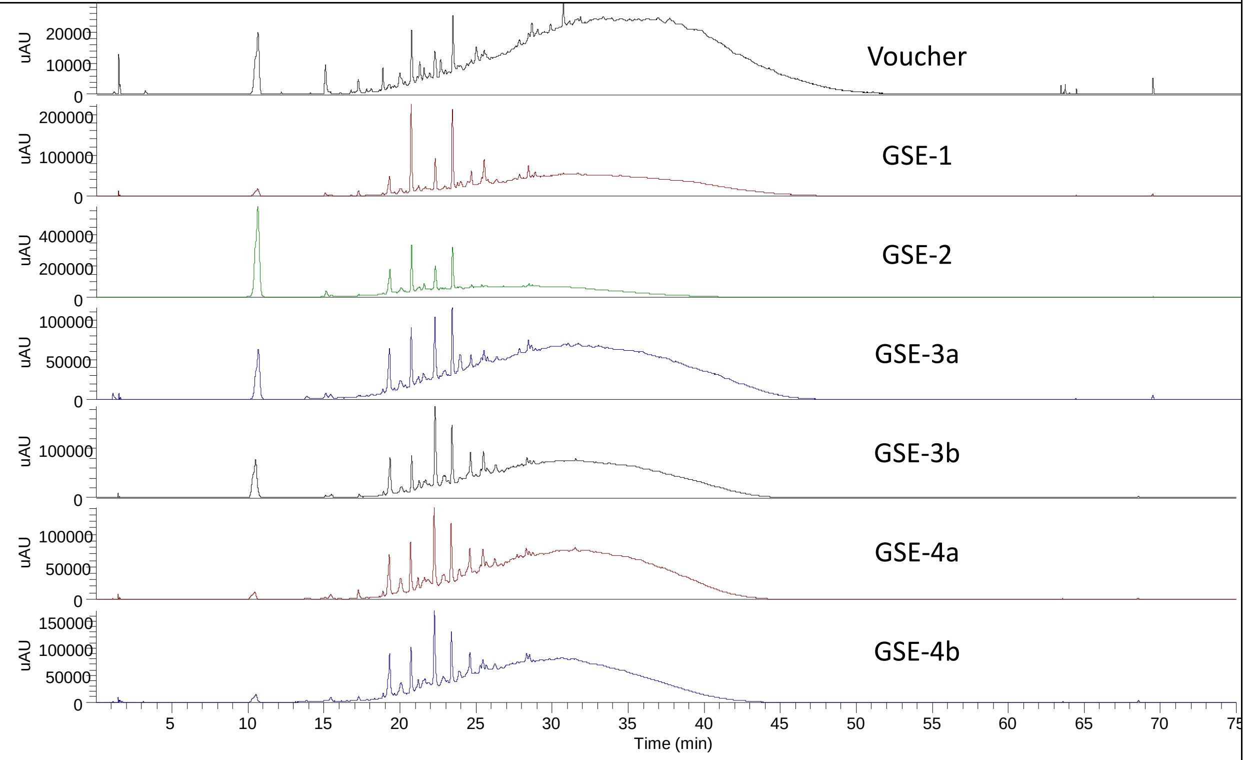

Figure S11. (A) The CAD and (B) UV (280 nm) chromatographic profiles for the authenticated GSE voucher and the various extracts from different suppliers.

Table S1. Proposed identifications of components producing CAD peaks in the UHPLC-UV-CAD-HRMS/MS analysis of grape seed (*Vitis vinifera*) extract for GSE-1.

CAD Peak	RT (min)	Exp. *m/z*<br>-, top<br>+, bottom	Mass Acc. (ppm)<br>-, top<br>+, bottom	Proposed ID<br>Molecular Formula	Structure	High Resolution Fragments [1]<br>(*m/z* intensity)
1a	1.04	158.9781<br>110.0084	3.441<br>-3.153	Magnesium salts	Mg^{2+}	
1b		174.9566<br>125.9859	2.788<br>-2.430	Calcium salts	Ca^{2+}	
2a	1.18	112.9858<br>190.9117	1.712<br>-1.381	Sodium salts	Na^{+}	
2b		128.9598<br>122.9243	1.832<br>-1.949	Potassium salts	K^{+}	
3a	1.37	179.0555<br>203.0524	-2.576<br>-1.178	Monosaccharide<br>$C_6H_{12}O_6$	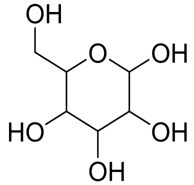	Negative<br>59.0139 (100), 71.0139 (42), 89.0243 (39), 101.0245 (7), 113.0243 (6)
3b		195.0502<br>219.0263 [6]	-3.927<br>-0.477	Gluconic Acid<br>$C_6H_{12}O_7$	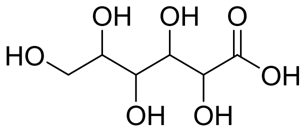	Negative<br>111.0089 (14), 129.0195 (98), 159.0301 (100), 177.0407 (12), 195.0510 (43)
3c		146.0456<br>148.0602	-3.435<br>-1.178	Glutamic Acid<br>$C_5H_9NO_4$	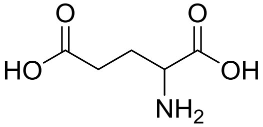	Positive<br>84.0444 (3), 102.0550 (14), 130.0500 (100)
3d		N/A<br>266.1594	N/A<br>-1.255	Choline hexoside<br>$C_{11}H_{23}NO_6$	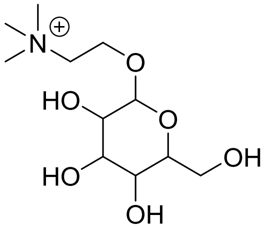	Positive<br>104.1070 (53), 266.1602 (100)<br><br>MS^3<br>60.0806 (100)
3e		N/A<br>337.1712	N/A<br>-1.796	Arginyl fructose<br>$C_{12}H_{24}N_4O_7$	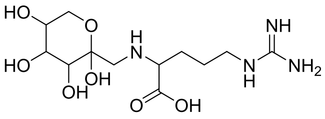	Positive<br>173.1402 (34), 175.1193 (54), 217.1301 (35), 257.1614 (100), 260.1133 (27), 283.1407 (), 301.1512 (18), 319.1617 (90)

CAD Peak	RT (min)	Exp. *m/z* -, top +, bottom	Mass Acc. (ppm) -, top +, bottom	Proposed ID Molecular Formula	Structure	High Resolution Fragments [1] (*m/z* intensity)
3f		N/A<br>325.1122	N/A<br>-1.917	Cellobiosan<br>$C_{12}H_{20}O_{10}$	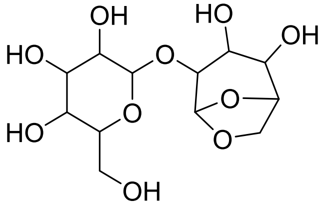	Positive<br>145.0497 (11), 253.0712 (14), 271.0824 (19), 289.0910 (24), 306.1184 (100), 307.1151 (94)
3g		N/A<br>118.0860	N/A<br>-2.923	Isovaline<br>$C_5H_{11}NO_2$	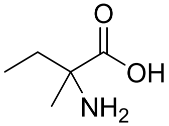	Positive<br>72.0807 (100), 101.0598 (15)
4a	1.53	149.0087<br>N/A	-3.028<br>N/A	Tartaric Acid<br>$C_4H_6O_6$	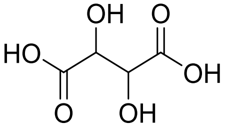	Negative<br>59.0140 (65), 87.0089 (100). 103.0038 (95), 130.9988 (42), 149.0094 (17)
4b		341.1073<br>365.1052 [6]	-4.880<br>-0.582	Disaccharide<br>$C_{12}H_{22}O_{11}$	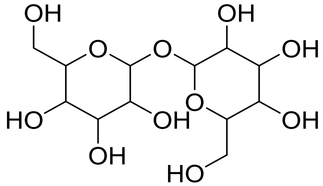	Negative<br>89.0246 (18), 113.0246 (23), 119.0352 (14), 143.0353 (26), 161.0459 (26), 179.0563 (100)
4c		N/A<br>116.0703	N/A<br>-2.456	Proline<br>$C_5H_9NO_2$	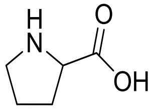	Positive<br>70.0651 (100)
4d		N/A<br>138.0546	N/A<br>-2.644	N-methylnicotinate<br>$C_7H_7NO_2$	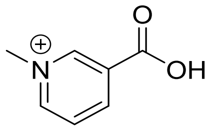	Positive<br>94.0649 (100)
5a	2.17	133.0139<br>157.0107 [6]	-2.380<br>-0.092	Malic Acid<br>$C_4H_6O_5$	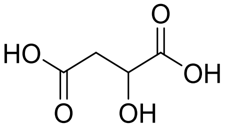	Negative<br>115.30037 (100)
5b		N/A<br>306.9972	N/A<br>N/A	Unidentified		Positive<br>172.9760 (4), 244.9968 (7), 270.9764 (8), 288.9866 (100)

CAD Peak	RT (min)	Exp. *m/z* -, top +, bottom	Mass Acc. (ppm) -, top +, bottom	Proposed ID Molecular Formula	Structure	High Resolution Fragments [1] (*m/z* intensity)
6a	4.46	191.0192<br>215.0162 [6]	-2.857<br>0.122	Citric Acid<br>$C_6H_8O_7$	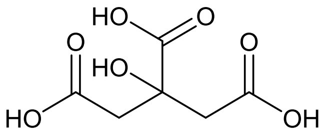	Negative<br>111.0089 (100), 173.0092 (13)<br>Positive<br>169.0110 (56), 197.0059 (100)
6b		128.0355<br>130.0498	1.747<br>0.122	Pyroglutamic Acid<br>$C_6H_8O_7$	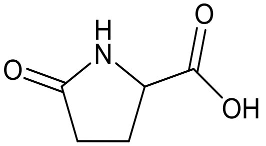	Positive<br>84.0444 (100)
7a	5.56	117.0189<br>N/A	-2.922<br>N/A	Succinic Acid<br>$C_4H_6O_4$	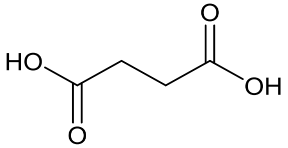	Negative<br>73.0296 (100), 99.0088 (53)
7b		180.0659<br>182.0807	-3.812<br>-2.470	Tyrosine<br>$C_9H_{11}NO_3$	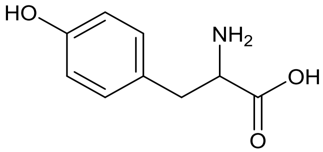	Positive<br>136.0758 (23),165.0547 (100)
7c		243.0611<br>N/A	-4.770<br>N/A	Uridine<br>$C_9H_{12}N_2O_6$	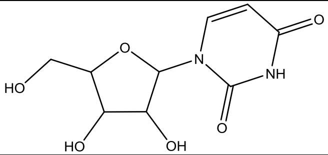	Negative<br>110.0249 (12), 140.0355 (4), 152.0356 (9), 174.9561 (6), 200.0566 (100)
7d		N/A<br>332.1329	N/A<br>-3.171	Glucopyranosyl pyridoxine<br>$C_{14}H_{21}NO_8$	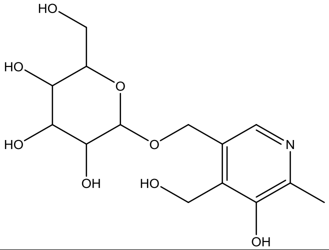	Positive<br>152.0708 (6), 170.0813 (7), 314.1236 (100)
7e		N/A<br>268.1032	N/A<br>-2.684	Adenosine<br>$C_{10}H_{14}N_5O_4$	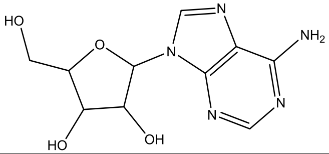	Positive<br>136.0618 (100)

CAD Peak	RT (min)	Exp. *m/z*<br>-, top<br>+, bottom	Mass Acc. (ppm)<br>-, top<br>+, bottom	Proposed ID<br>Molecular Formula	Structure	High Resolution Fragments [1]<br>(*m/z* intensity)
7f		N/A<br>294.1537	N/A<br>-3.972	Leucine-Fructose<br>$C_{12}H_{23}NO_7$	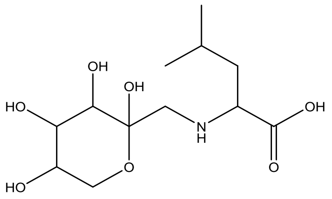	Positive<br>276.1445 (100)<br><br>MS^3<br>132.1021 (12), 230.1390 (44), 248.1495 (26), 258.1339 (100)
7g		251.0760<br>N/A	-4.782<br>N/A	Unidentified<br>$C_9H_{16}O_8$		Negative<br>151.0612 (100), 204.9751 (6), 232.0472 (7)
7h		253.0916<br>N/A	-4.705<br>N/A	Unidentified<br>$C_9H_{18}O_8$		Negative<br>207.0874 (100)
7i		289.0663<br>N/A	-4.873<br>N/A	Unidentified<br>$C_{10}H_{14}O_8N_2$		Negative<br>174.9561 (55), 188.9494 (97), 221.0669 (100), 243.0623 (75), 271.0947 (23)
7j		N/A<br>229.1541	N/A<br>-2.440	Unidentified<br>$C_{11}H_{21}N_2O_3$		Positive<br>70.0648 (5), 124.0754 (8), 142.0867 (100), 170.0813 (8), 211.1444 (3)
7k		N/A<br>241.1541	N/A<br>-2.318	Unidentified<br>$C_{12}H_{21}N_2O_3$		Positive<br>84.0807 (6), 128.1071 (24), 144.1022 (15), 194.0814 (29), 196.0970 (100), 213.1601 (5)
7l		N/A<br>314.0909	N/A<br>-4.658	Unidentified<br>$C_{19}H_{12}N_3O_2$		Positive<br>97.0284 (8), 136.0620 (100), 143.0165 (4), 161.0269 (3), 179.0376 (8), 296.0817 (92)
8	9.46	169.0137<br>171.0288	-3.234<br>-0.057	Gallic acid<br>$C_7H_6O_5$	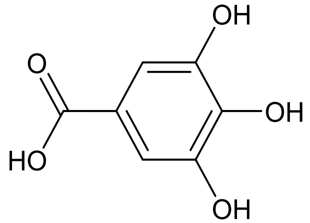	Negative<br>125.0245 (100), 169.0143 (25)
9a	13.39	331.0655<br>333.0815	-4.863<br>-0.520	Glucogallin<br>$C_{13}H_{16}O_{10}$	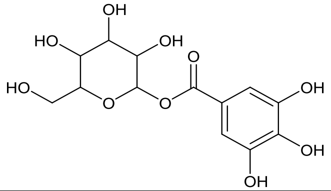	Negative<br>169.0142 (100)<br><br>MS^3<br>125.0246 (100)

CAD Peak	RT (min)	Exp. *m/z*<br>-, top<br>+, bottom	Mass Acc. (ppm)<br>-, top<br>+, bottom	Proposed ID<br>Molecular Formula	Structure	High Resolution Fragments [(1)]<br>(*m/z* intensity)
9b		N/A<br>206.5312 [(5)]	N/A<br>N/A	Unidentified		Positive<br>186.0182 (42), 195.0235 (100), 204.0289 (25)
10	16.39	203.0818<br>205.0969	-3.698<br>-1.337	Tryptophan<br>$C_{11}H_{12}N_2O_2$	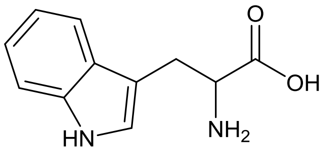	Negative<br>116.0507 (32), 142.0663 (11),<br>159.0930 (100), 186.056 (12)<br><br>Positive<br>188.0706 (100)
11	18.62	577.1350<br>579.1482	-0.293<br>-2.543	Procyanidin B1<br>$C_{30}H_{26}O_{12}$	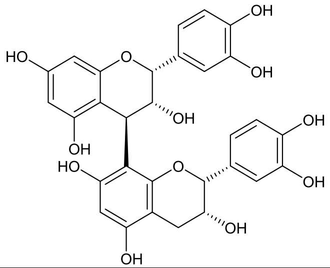	Negative<br>289.0719 (40), 407.0774 (76), 408.0805 (14),<br>425.0875 (100), 451.1033 (42), 559.1244 (10)
12	19.02	577.1347<br>579.1485	-0.848<br>-2.077	Procyanidin B<br>$C_{30}H_{26}O_{12}$	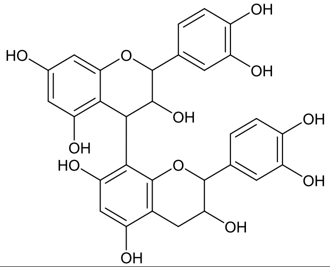	Negative<br>269.0455 (17), 289.0719 (24), 407.0774 (40),<br>425.0875 (100), 439.1030 (42),<br>451.1033 (21), 533.1451 (21), 559.1244 (23)
13	19.57	577.1346<br>579.1479	-0.865<br>-3.130	Procyanidin B<br>$C_{30}H_{26}O_{12}$	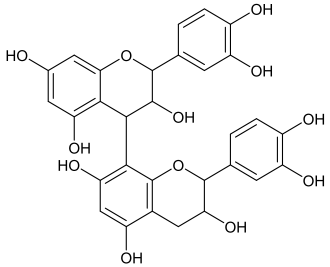	Negative<br>289.0719 (40), 407.0774 (76), 408.0805 (14),<br>425.0875 (100), 451.1033 (42), 559.1244 (10)

CAD Peak	RT (min)	Exp. *m/z* -, top +, bottom	Mass Acc. (ppm) -, top +, bottom	Proposed ID Molecular Formula	Structure	High Resolution Fragments [1] (*m/z* intensity)
14	20.25	289.0710<br>291.0855	-2.530<br>-2.867	Catechin<br>$C_{15}H_{14}O_6$	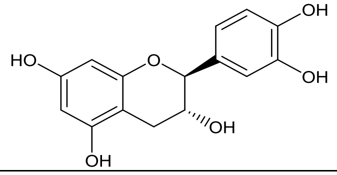	Negative<br>179.0351 (15), 205.0505 (34), 245.0816 (100)
15a	20.57	577.1346<br>579.1479	-0.917<br>-3.026	Procyanidin B<br>$C_{30}H_{26}O_{12}$	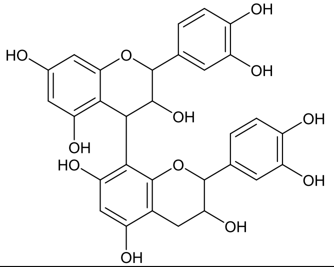	Negative<br>289.0719 (40), 407.0774 (76), 408.0805 (14), 425.0875 (100), 451.1033 (42), 559.1244 (10)
15b		457.0776<br>459.0912	-0.076<br>-3.633	Gallocatechin Gallate isomer<br>$C_{22}H_{18}O_{11}$	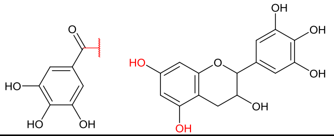	Negative<br>166.9990 (15), 289.0718 (100), 413.0877 (17)
15c		N/A<br>201.5167 [5]	N/A<br>N/A	Unknown		
16	20.69	865.1965<br>867.2111	-2.320<br>-2.272	Procyanidin C<br>$C_{45}H_{38}O_{18}$	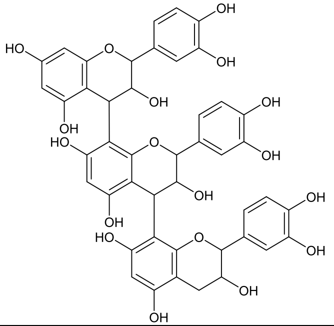	Negative<br>287.0569 (24), 407.0776 (38), 425.0890 (24), 543.0941 (20), 577.1364 (63), 587.1206 (24), 695.1427 (100), 713.1527 (31), 739.1671 (41), 847.1898 (14), 867.2060 (53)
17	21.01	401.1453<br>425.1408 [6]	0.049<br>-2.301	Benzyl alcohol<br>$C_{18}H_{26}O_{10}$	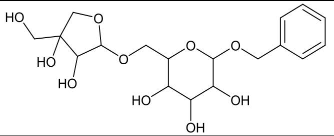	Negative<br>161.0454 (12), 269.1026 (100)

CAD Peak	RT (min)	Exp. *m/z*<br>-, top<br>+, bottom	Mass Acc. (ppm)<br>-, top<br>+, bottom	Proposed ID<br>Molecular Formula	Structure	High Resolution Fragments [1]<br>(*m/z* intensity)
18	21.14	652.1323 [5]<br>N/A	-0.803<br>N/A	Galloylated Procyanidin (tetramer)	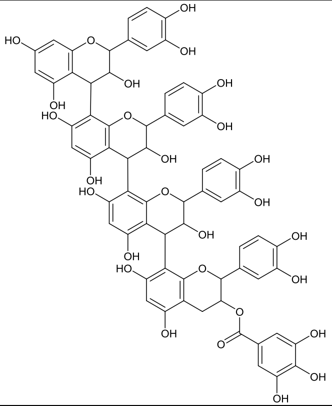	Negative<br>289.0722 (43), 303.0516 (16), 407.0780 (8), 491.0976 (11), 567.1045 (23), 576.1116 (93), 727.1305 (44), 853.1628 (13), 1015.1936 (54), 1179.2414 (13)
19	21.82	577.1350<br>579.1485	-0.207<br>-2.111	Procyanidin B2<br>$C_{30}H_{26}O_{12}$	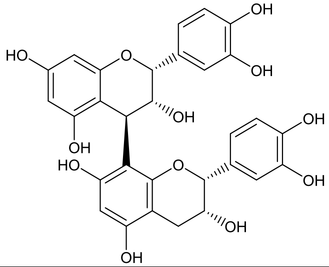	Negative<br>289.0719 (40), 407.0774 (76), 408.0805 (14), 425.0875 (100), 451.1033 (42), 559.1244 (10)
20a	22.39	577.1344<br>579.1486	-1.229<br>-1.973	Procyanidin B<br>$C_{30}H_{26}O_{12}$	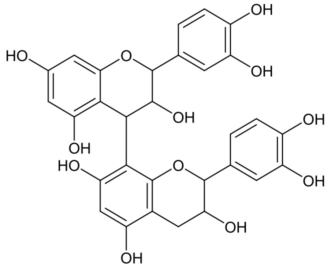	Negative<br>269.0455 (17), 289.0719 (24), 407.0774 (40), 425.0875 (100), 439.1030 (42), 451.1033 (21), 533.1451 (21), 559.1244 (23)
20b		521.2028<br>545.1978 [6]	-0.144<br>-2.903	Lariciresinol Glucoside analogue<br>$C_{26}H_{34}O_{11}$	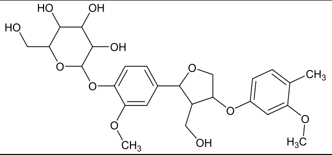	Negative<br>359.1492 (100)
20c		N/A<br>219.1010	N/A<br>-2.468	$C_{13}H_{14}O_3$		Positive<br>131.0858 (3), 159.0804 (14), 189.0911 (100), 191.1069 (16), 201.0913 (32)

CAD Peak	RT (min)	Exp. *m/z* -, top +, bottom	Mass Acc. (ppm) -, top +, bottom	Proposed ID Molecular Formula	Structure	High Resolution Fragments [1] (*m/z* intensity)
21	22.73	577.1341<br>579.1487	-1.801<br>-1.766	Procyanidin B<br>$C_{30}H_{26}O_{12}$	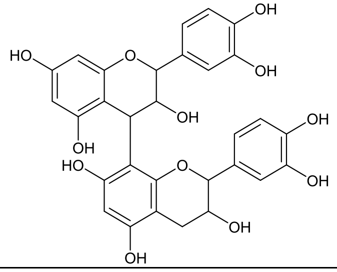	Negative<br>269.0455 (17), 289.0719 (24), 407.0774 (40), 425.0875 (100), 439.1030 (42), 451.1033 (21), 533.1451 (21), 559.1244 (23)
22	23.02	289.0714<br>291.0857	-1.389<br>-2.180	Epicatechin<br>$C_{15}H_{14}O_6$	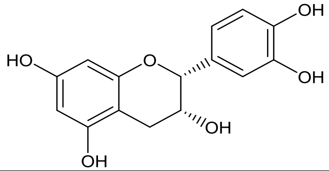	Negative<br>179.0351 (15), 205.0505 (34), 245.0816 (100)
23a	23.36	291.0873<br>293.1014	-0.211<br>-1.995	1-(3',4'-dihydroxy-phenyl)-3-(2",4",6"-trihydroxyophenyl) propan-2-ol<br>$C_{15}H_{16}O_6$	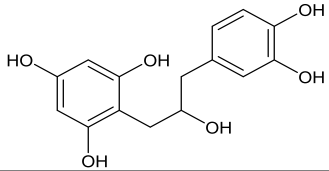	Negative<br>123.0454 (10), 167.0351 (20), 205.0870 (13), 247.0974 (100), 291.0871 (13)
23b		449.1088<br>473.1045 [6]	-0.344<br>-1.929	Dihydrokaempferol 3-O-ß-D-glucoside<br>$C_{21}H_{22}O_{11}$	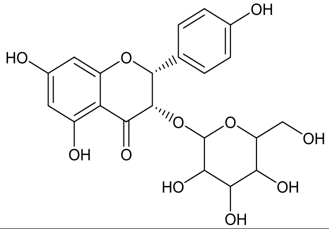	Negative<br>259.0614 (35), 269.0458 (27), 287.0560 (100)
24a	23.54	457.0777<br>459.0912	0.078<br>-2.239	Epigallocatechin Gallate<br>$C_{22}H_{18}O_{11}$	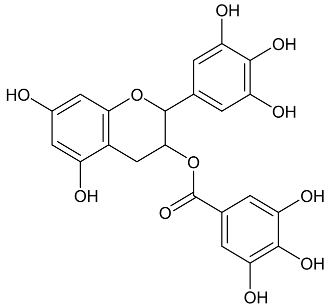	Negative<br>169.0143 (100), 287.0559 (11), 305.0663 (34), 331.0454 (56)

CAD Peak	RT (min)	Exp. *m/z* -, top +, bottom	Mass Acc. (ppm) -, top +, bottom	Proposed ID Molecular Formula	Structure	High Resolution Fragments [1] (*m/z* intensity)
24b		729.1448<br>731.1596	-1.725<br>-1.438	Galloylated Procyanidin (dimer)<br>$C_{37}H_{30}O_{16}$	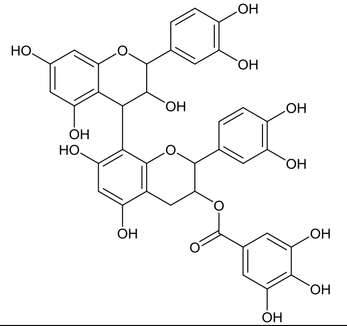	Negative<br>407.0783 (34), 425.0890 (25), 559.1271 (37), 577.1355 (100)
24c		576.1269 [5]<br>1155.2740	-0.684<br>-2.102	Procyanidin (tetramer)<br>$C_{60}H_{50}O_{24}$	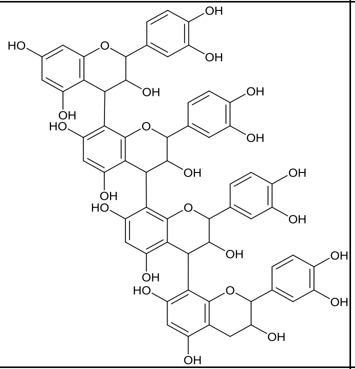	Negative<br>287.0561 (24), 289.0715 (40), 449.0869 (15), 491.0986 (76), 500.1021 (100), 567.1210 (17), 701.1504 (11), 739.1631 (16), 863.1813 (47), 1027.2266 (33)
25	23.99	865.1970<br>867.2120	-1.765<br>-1.211	Procyanidin C<br>$C_{45}H_{38}O_{18}$	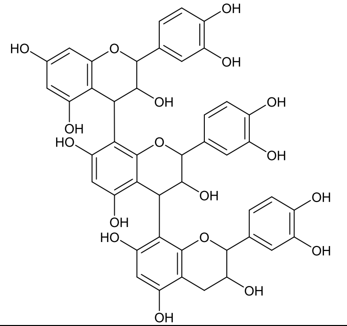	Negative<br>287.0569 (24), 407.0776 (38), 425.0890 (24), 543.0941 (20), 577.1364 (63), 587.1206 (24), 695.1427 (100), 713.1527 (31), 739.1671 (41), 847.1898 (14), 867.2060 (53)

CAD Peak	RT (min)	Exp. *m/z* -, top +, bottom	Mass Acc. (ppm) -, top +, bottom	Proposed ID Molecular Formula	Structure	High Resolution Fragments [1] (*m/z* intensity)
26a	24.24	865.1968<br>867.2114	-1.962<br>-1.996	Procyanidin C1<br>$C_{45}H_{38}O_{18}$	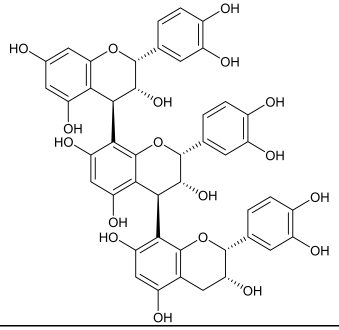	Negative<br>287.0569 (24), 407.0776 (38), 425.0890 (24), 543.0941 (20), 577.1364 (63), 587.1206 (24), 695.1427 (100), 713.1527 (31), 739.1671 (41), 847.1898 (14), 867.2060 (53)
26b		515.1920<br>539.1871 [6]	-0.505<br>-3.150	Leptolepisol D<br>$C_{27}H_{32}O_{10}$	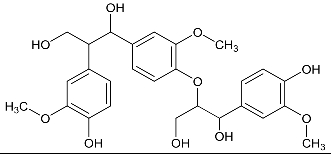	Negative<br>195.0664 (18), 271.0972 (100), 319.1184 (51), 467.1702 (64)
27a	24.94	577.1349<br>579.1483	-0.432<br>-2.387	Procyanidin B<br>$C_{30}H_{26}O_{12}$	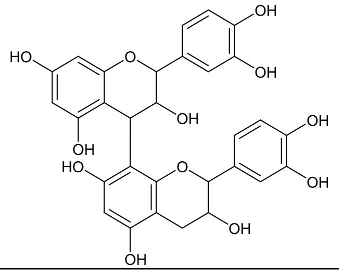	Negative<br>289.0719 (40), 407.0774 (76), 408.0805 (14), 425.0875 (100), 451.1033 (42), 559.1244 (10
27b		508.1011 [5]<br>1019.2260	-0.117<br>1.865	Galloylated Procyanidin (trimer)<br>$C_{52}H_{42}O_{22}$	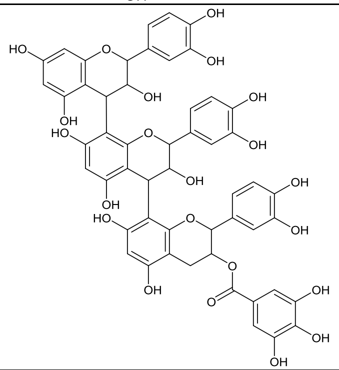	Negative<br>287.0562 (21), 423.0721 (100), 441.0822 (41), 575.1195 (23), 603.1149 (16), 729.1450 (36), 847.1861 (16), 891.1776 (52)

CAD Peak	RT (min)	Exp. *m/z* -, top +, bottom	Mass Acc. (ppm) -, top +, bottom	Proposed ID Molecular Formula	Structure	High Resolution Fragments [1] (*m/z* intensity)
28a	25.07	729.1451<br>731.1588	-1.341<br>-2.532	Galloylated Procyanidin (dimer)<br>$C_{37}H_{30}O_{16}$	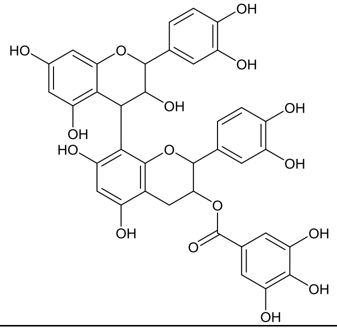	Negative<br>289.0721 (30), 407.0775 (100), 441.0830 (39), 451.1035 (52), 559.0978 (30), 577.1410 (43), 603.1148 (47), 731.1519 (33)
28b		576.1270 [5]<br>1155.2772	-0.511<br>0.642	Procyanidin (tetramer)<br>$C_{60}H_{50}O_{24}$	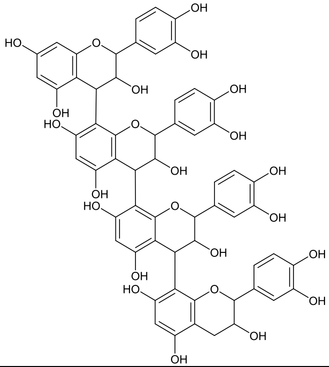	Negative<br>287.0569 (33), 289.0720 (100), 407.0773 (57), 425.0876 (70), 451.1038 (50), 491.0986 (38), 500.1041 (62), 559.1296 (17), 863.1825 (56)
29a	25.30	508.1010 [5]<br>1019.2204	-0.157<br>-3.580	Galloylated Procyanidin (trimer)<br>$C_{52}H_{42}O_{22}$	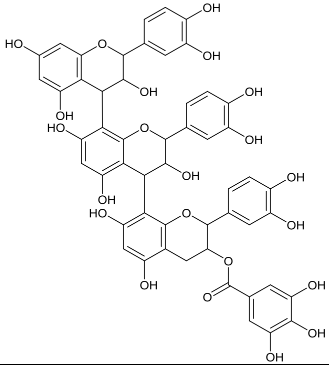	Negative<br>289.0716 (39), 303.0511 (100), 423.0713 (16),432.0923 (51), 577.1347 (30), 695.1393 (52), 713.1497 (40), 727.1293 (30)

CAD Peak	RT (min)	Exp. *m/z*<br>-, top<br>+, bottom	Mass Acc. (ppm)<br>-, top<br>+, bottom	Proposed ID<br>Molecular Formula	Structure	High Resolution Fragments [1]<br>(*m/z* intensity)
29b		521.2027<br>545.1981 [6]	-0.182<br>-2.335	Lariciresinol Glucosides analogue<br>$C_{26}H_{34}O_{11}$	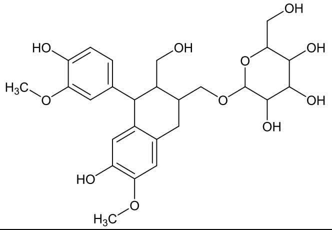	Negative<br>359.1489 (100)
30a	25.91	508.1007 [5]<br>1019.2219	-0.767<br>-2.148	Galloylated Procyanidin (trimer)<br>$C_{52}H_{42}O_{22}$	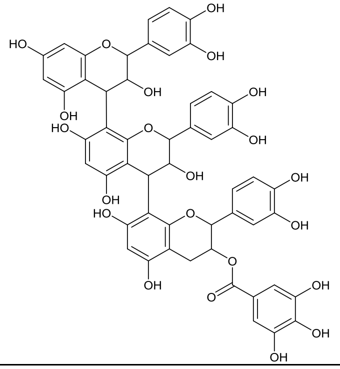	Negative<br>289.0716 (39), 303.0511 (100), 423.0713 (16),432.0923 (51), 577.1347 (30), 695.1393 (52), 713.1497 (40), 727.1293 (30)
30b		720.1577 [5]<br>N/A	-1.829<br>N/A	Procyanidin (pentamer)<br>$C_{75}H_{62}O_{30}$	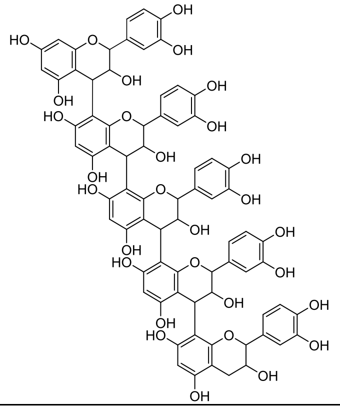	Negative<br>289.0722 (52), 407.0778 (22), 559.1128 (25), 577.1348 (78), 635.6320 (100), 863.1835 (61), 865.1957 (51), 1153.2533 (33), 1316.2962 (28)

CAD Peak	RT (min)	Exp. *m/z* -, top +, bottom	Mass Acc. (ppm) -, top +, bottom	Proposed ID Molecular Formula	Structure	High Resolution Fragments [1] (*m/z* intensity)
31	26.32	577.1347<br>579.1481	-0.848<br>-2.715	Procyanidin B<br>$C_{30}H_{26}O_{12}$	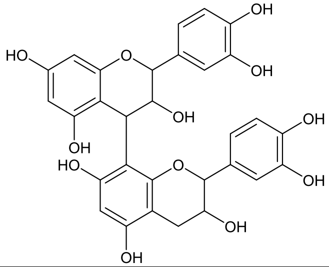	Negative<br>269.0455 (17), 289.0719 (24), 407.0774 (40), 425.0875 (100), 439.1030 (42), 451.1033 (21), 533.1451 (21), 559.1244 (23)
32a	27.37	508.1008 [5]<br>1019.2223	-0.649<br>-1.726	Galloylated Procyanidin (trimer)<br>$C_{52}H_{42}O_{22}$	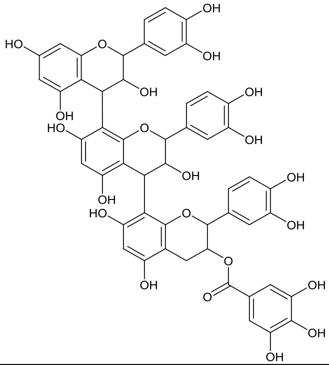	Negative<br>289.0716 (39), 303.0511 (100), 423.0713 (16),432.0923 (51), 577.1347 (30), 695.1393 (52), 713.1497 (40), 727.1293 (30)
32b		609.1455<br>611.1591	-1.031<br>-2.620	Rutin<br>$C_{27}H_{30}O_{16}$	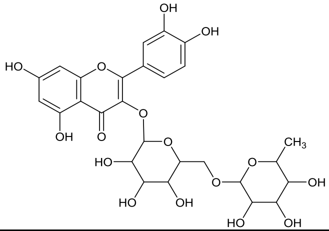	Negative<br>301.0349 (100), 343.0453 (7)<br>Positive<br>303.0500 (100), 465.1027 (29)
32c		881.1556<br>883.1705	-1.653<br>-1.302	Digalloylated Procyanidin (dimer)<br>$C_{44}H_{34}O_{20}$	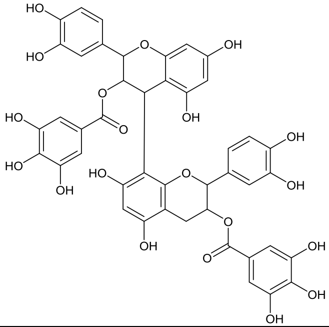	Negative<br>407.0778 (22), 441.0830 (6), 559.1328 (14), 603.1153 (6), 711.1359 (23), 729.1461 (100)

CAD Peak	RT (min)	Exp. *m/z* -, top +, bottom	Mass Acc. (ppm) -, top +, bottom	Proposed ID Molecular Formula	Structure	High Resolution Fragments [(1)] (*m/z* intensity)
32d		579.1505<br>581.1642	-0.448<br>-2.018	2-(3,4-dihydroxy-phenyl)-4-(3-(3-(3,4-dihydroxyphenyl)-2-hydroxypropyl)-2,4,6-trihydroxylphenyl) chromane-3,5,7-triol<br>$C_{30}H_{28}O_{12}$	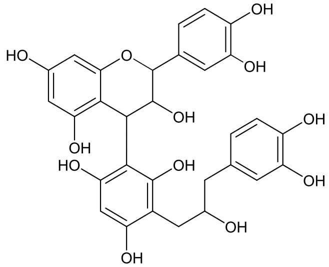	Negative<br>287.0562 (5), 291.0871 (58), 409.0922 (100), 427.1028 (34), 453.1182 (59), 561.1400 (34)
32e		591.1507<br>593.1643	-0.202<br>-1.758	Procyanidin B-type analogue<br>$C_{31}H_{28}O_{12}$	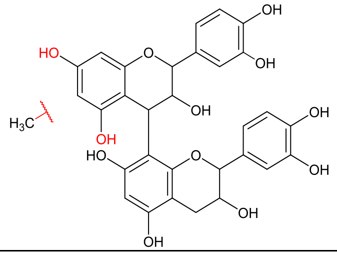	Negative<br>215.0716 (20), 257.0822 (9), 289.0720 (74), 301.0720 (81), 439.1037 (100), 547.1598 (14)
33a	28.00	441.0826<br>443.0961	-0.272<br>-2.625	Epicatechin gallate<br>$C_{22}H_{18}O_{10}$	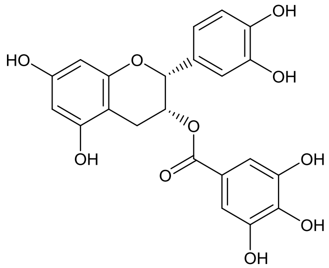	Negative<br>169.0144 (17), 271.0609 (10), 289.0713 (100), 331.0454 (14)
33b		577.1347<br>579.1481	-0.831<br>-2.750	Procyanidin B<br>$C_{30}H_{26}O_{12}$	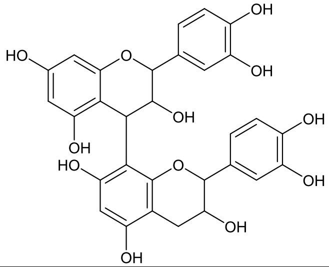	Negative<br>289.0719 (40), 407.0774 (76), 408.0805 (14), 425.0875 (100), 451.1033 (42), 559.1244 (10

CAD Peak	RT (min)	Exp. *m/z* -, top +, bottom	Mass Acc. (ppm) -, top +, bottom	Proposed ID Molecular Formula	Structure	High Resolution Fragments [(1)] (*m/z* intensity)
34a	28.41	441.0826<br>443.0959	-0.227<br>-3.054	Catechin gallate<br>$C_{22}H_{18}O_{10}$	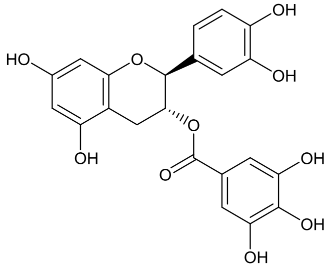	Negative<br>169.0144 (17), 271.0609 (10), 289.0713 (100), 331.0454 (14)
34b		508.1008 [(5)]<br>1019.227	-0.531<br>-1.304	Galloylated Procyanidin (trimer)<br>$C_{52}H_{42}O_{22}$	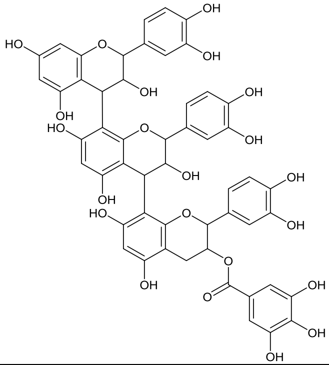	Negative<br>287.0561 (18), 413.0878 (14), 423.0720 (33), 432.0775 (100), 499.0954 (10), 575.1191 (22), 729.1450 (40), 891.1760 (47)
34c		459.1267<br>461.1428	-3.993<br>-3.043	Embigenin<br>$C_{23}H_{24}O_{10}$	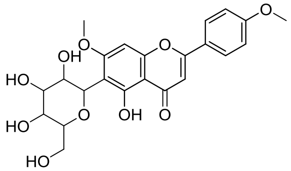	Positive<br>323.0907 (4), 341.1022 (23), 365.1027 (11), 395.1131 (27), 407.1127 (8), 425.1236 (28), 443.1341 (100)
35	66.70	455.3526<br>457.3665	-1.139<br>-2.475	Ursolic Acid<br>$C_{30}H_{48}O_3$	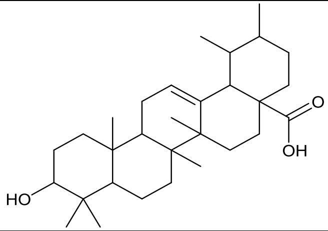	Negative<br>407.3318 (100)<br>Positive<br>393.3510 (7), 411.3625 (100), 439.3576 (69)
36	67.04	279.2329<br>281.2470	-0.048<br>-1.873	Linoleic Acid<br>$C_{18}H_{32}O_2$	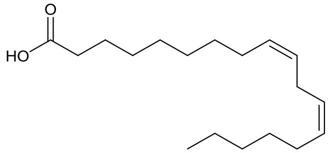	Negative<br>261.2222 (100)

CAD Peak	RT (min)	Exp. *m/z*<br>-, top<br>+, bottom	Mass Acc. (ppm)<br>-, top<br>+, bottom	Proposed ID<br>Molecular Formula	Structure	High Resolution Fragments (1)<br>(*m/z* intensity)
37	68.37	255.2332<br>257.2470	0.809<br>-1.931	Palmitic Acid<br>$C_{16}H_{32}O_2$	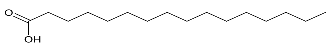	Negative<br>237.2222 (100)
38	68.66	281.2488<br>283.2626	0.627<br>-1.860	Oleic Acid<br>$C_{18}H_{34}O_2$	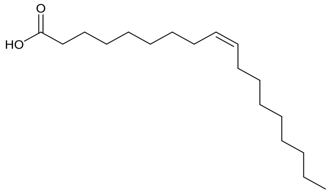	Negative<br>263.2378 (100)<br>Positive<br>185.1538 (6), 199.1696 (8), 213.1852 (8), 227.2011 (6), 247.2423 (26), 265.2528 (100)
39	15-45	-	-	Tannins	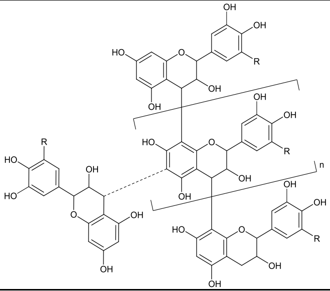	

(1) High resolution MS/MS experiments were performed using CID with a NCE of 30.

Table S2. Proposed identifications of components producing FID peaks in the GC-FID and GC-HRMS analysis of grape seed (*Vitis vinifera*) extract for GSE-1.

Peak #	Kovats Retention Index	Exp. *m/z* (1) Mass Accuracy (ppm)	ID Molecular Formula	Structure	High Resolution Fragments (2) (*m/z*)
1	732	87.0441 (-0.006)	*Unidentified* $C_4H_6O_2$		61.0284, 43.0178, 33.0335 45.0335, 42.0099
2	894	91.0390 (0.192)	Dihydroxyacetone $C_3H_6O_3$	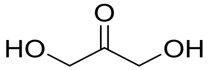	42.0098, 43.0177, 31.0179, 33.0335
3	966	91.0390 (0.039)	Glycerol $C_3H_8O_3$	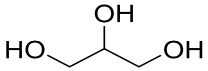	61.0284, 43.0177, 44.0257
4	1188	111.0441 (0.216)	Catechol $C_6H_6O_2$	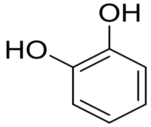	110.0362, 81.0335, 82.0413, 63.0229, 64.0308, 92.0257
5	1284	125.0597 (-0.288)	4-methyl catechol $C_7H_8O_2$	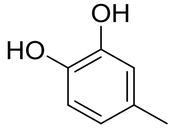	124.0519, 78.0464, 43.0178, 95.0492, 106.0414, 107.0492
6	1362	127.0390 (-0.084)	1,2,3-benzenetriol $C_6H_6O_3$	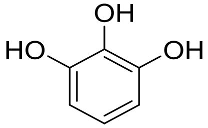	126.0310, 108.0205, 80.0256, 97.0284, 50.0151, 39.0227
7	1593	153.0543 (-1.768)	*Unidentified* $C_8H_8O_3$		123.0440, 152.0468, 77.0389 105.0336, 95.0492
8	1801	165.0544 (-1.579)	*Unidentified* $C_9H_8O_3$		136.0520, 164.0468, 89.0386, 107.0492, 43.0177, 79.0543

(1) Experimental *m/z* was acquired by chemical ionization (CI) on a high resolution mass spectrometer. The values indicate $[M+H]^+$.
(2) Fragmentation was acquired by electron ionization (EI) on a high resolution mass spectrometer.
